# Uracil-DNA glycosylase efficiency is modulated by substrate rigidity

**DOI:** 10.1038/s41598-023-30620-0

**Published:** 2023-03-08

**Authors:** Paul B. Orndorff, Souvik Poddar, Aerial M. Owens, Nikita Kumari, Bryan T. Ugaz, Samrat Amin, Wade D. Van Horn, Arjan van der Vaart, Marcia Levitus

**Affiliations:** 1 Department of Chemistry, University of South Florida, Tampa, FL 33620 USA; 2 School of Molecular Sciences, Arizona State University, Tempe, AZ 85287 USA; 3 The Biodesign Institute Center for Single Molecule Biophysics, Arizona State University, Tempe, AZ 85287 USA; 4 The Biodesign Institute Virginia G. Piper Center for Personalized Diagnostics, Arizona State University, Tempe, AZ 85287 USA; 5 Magnetic Resonance Research Center, Arizona State University, Tempe, AZ 85287 USA

**Keywords:** DNA, Biophysical chemistry, DNA repair enzymes

## Abstract

Uracil DNA-glycosylase (UNG) is a DNA repair enzyme that removes the highly mutagenic uracil lesion from DNA using a base flipping mechanism. Although this enzyme has evolved to remove uracil from diverse sequence contexts, UNG excision efficiency depends on DNA sequence. To provide the molecular basis for rationalizing UNG substrate preferences, we used time-resolved fluorescence spectroscopy, NMR imino proton exchange measurements, and molecular dynamics simulations to measure UNG specificity constants (*k*_cat_/*K*_M_) and DNA flexibilities for DNA substrates containing central AUT, TUA, AUA, and TUT motifs. Our study shows that UNG efficiency is dictated by the intrinsic deformability around the lesion, establishes a direct relationship between substrate flexibility modes and UNG efficiency, and shows that bases immediately adjacent to the uracil are allosterically coupled and have the greatest impact on substrate flexibility and UNG activity. The finding that substrate flexibility controls UNG efficiency is likely significant for other repair enzymes and has major implications for the understanding of mutation hotspot genesis, molecular evolution, and base editing.

## Introduction

Cellular repair pathways maintain genetic integrity against thousands of daily DNA lesions. The repair of small, non-helix distorting lesions, is initiated by specialized DNA glycosylases that catalyze the excision of the damaged base^1^. DNA sequence effects have been identified for many glycosylases and alkyl-transferases that repair base mismatches, uracil bases, alkylated bases, etc^2–7^. However, the molecular features that give rise to preferences for particular DNA sequences have not yet been resolved. Here, we focus on understanding how DNA sequence impacts the repair of uracil by uracil-DNA glycosylase (UNG). Uracil is a highly mutagenic and common lesion in DNA that arises from dUTP misincorporation or from spontaneous cytosine deamination^8^. Unrepaired cytosine deamination results in U:G mismatches that give rise to G:C to A:T transition mutations^9^.

The crystal structure of the human UNG-DNA complex shows the uracil extruded from the duplex into the enzyme active site^10^. The first step in uracil recognition by UNG relies on trapping spontaneous extrahelical excursions of uracil^11–13^, which occur at enhanced rates compared to thymine in the same sequence context^14^. Consistent with this observation, a thermodynamic study of the effect of thymine to uracil substitutions found reduced base stacking in U:A base pairs compared to T:A^15^. While these studies provide a molecular framework for understanding how UNG distinguishes between uracil and thymine, the molecular principles that determine UNG substrate preferences are still poorly understood. In *E. coli*, the efficiency of UNG varies more than 15-fold depending on the DNA context^4,5^. A vicinal thymine 3′ of uracil generally results in poor excision, and substrates with high local GC content are generally poor substrates^16,17^. Yet, the underlying principles that dictate UNG substrate preferences still remain elusive.

Preferential excision of uracil does not correlate with DNA melting temperatures; for instance, substrates containing uracil in TUA contexts are better UNG substrates than sequences with AUT contexts^4,5^. The differences between TUA and AUT sequences are remarkable and are reminiscent of the asymmetries observed in the mechanical properties of undamaged DNA. TA steps are particularly flexible with regard to roll, slide, and twist, while AT steps are more rigid^18,19^. DNA flexibility is indeed suspected to dictate the binding preferences of various non-specific DNA enzymes and proteins. For instance, DNA flexibility explains sequence preferences in phosphodiester backbone cleavage by endonuclease DNase 1^20^, and the role of DNA sequence in nucleosome stability and dynamics^21^. In this work, we test the hypothesis that UNG activity is dictated by the intrinsic local deformability of the DNA sequence around the uracil. Structural studies provide a rationale for this hypothesis; the formation of the catalytically active UNG-DNA complex requires DNA hydrogen bond breakage and loss of stabilizing base-stacking interactions^10,22^, and because these interactions are determined by the mechanical properties of DNA, the intrinsic deformability of the region surrounding the lesion is likely an important variable in understanding catalytic efficiency. A previous study that compared substrates containing uracil in AUA and GUG contexts hinted at a connection between substrate flexibility and UNG activity^23^, but the small scale of the study (2 substrates) was not sufficient to establish a correlation between the two variables, nor to determine the molecular basis for substrate preference.

To illuminate the link between UNG repair efficiency and substrate flexibility, we determined UNG specificity constants (*k*_*cat*_/*K*_M_) for a variety of designed substrates and correlated the outcomes with the results of biophysical experiments and MD simulations that probe DNA substrate flexibility. Our results not only establish a clear link between the fundamental nature of UNG substrate flexibility, the contributions of distinct substrate dynamics, and the hierarchy of these motions in regulating the resulting UNG catalytic efficiency, but also identify and quantify allosteric coupling of bases flanking the uracil.

## Materials and methods

### Enzymes, oligonucleotides, and reagents

Uracil-DNA Glycosylase (UNG, MW = 25.7 kDa) was purchased from New England BioLabs (Catalog # M0280L) at a concentration of 5000 U/mL. Free 2-aminopurine riboside was purchased from AstaTech (PA, USA). Oligonucleotides containing canonical bases, uracil, or 2-aminopurine (2AP) were purchased from IDT (IA, USA) as desalted oligonucleotides. All DNA sequences are listed in Table 1 and are represented by a number and two letters. The number refers to the different DNA series (1–4) and the letters refer to the bases adjacent to the uracil. For instance, the four substrates in series 1 (1TA, 1TT, 1AA and 1AT) share the same overall sequence but differ in the bases adjacent to the uracil. Oligonucleotides for fluorescence experiments (kinetic assays, time-resolved fluorescence, and fluorescence quantum yields) were 39 nt in length (Table 1). Oligonucleotides for NMR experiments were shortened to the central 13 nucleotides to facilitate resonance assignments while still ensuring duplex formation. All oligonucleotides were solubilized in 1 × PBS buffer (20 mM sodium phosphate, 100 mM NaCl, 50 µM EDTA, pH 7.0). Concentrations were determined from measured absorbances at 260 nm using extinction coefficients provided by IDT.

**Table 1:** DNA *s*equences.

Name	Motif^a^	Fluorescence/Kinetics^b^	NMR	Simulation
1TA	5′ T_1_G_2_C_3_A_4_**T**_**5**_**U**_**6**_**A**_**7**_A_8_T_9_G_10_A_11_C_12_ 3′	5′ L_1_ Motif R_1_ 3′	5′ G Motif 3′	5′ CG Motif G 3′
1TT	5′ T_1_G_2_C_3_A_4_**T**_**5**_**U**_**6**_**T**_**7**_A_8_T_9_G_10_A_11_C_12_ 3′	5′ L_1_ Motif R_1_ 3′	5′ G Motif 3′	5′ CG Motif G 3′
1AT	5′ T_1_G_2_C_3_A_4_**A**_**5**_**U**_**6**_**T**_**7**_A_8_T_9_G_10_A_11_C_12_ 3′	5′ L_2_ Motif R_1_ 3′	5′ G Motif 3′	5′ CG Motif G 3′
1AA	5′ T_1_G_2_C_3_A_4_**A**_**5**_**U**_**6**_**A**_**7**_A_8_T_9_G_10_A_11_C_12_ 3′	5′ L_2_ Motif R_1_ 3′	5′ G Motif 3′	5′ CG Motif G 3′
2TA	5′ T_1_G_2_A_3_A_4_**T**_**5**_**U**_**6**_**A**_**7**_G_8_T_9_T_10_A_11_C_12_ 3′	5′ L_3_ Motif R_2_ 3′	5′ G Motif 3′	5′ CG Motif G 3′
2TT	5′ T_1_G_2_A_3_A_4_**T**_**5**_**U**_**6**_**T**_**7**_G_8_T_9_T_10_A_11_C_12_ 3′	5′ L_3_ Motif R_1_ 3′	5′ G Motif 3′	5′ CG Motif G 3′
2AT	5′ T_1_G_2_A_3_A_4_**A**_**5**_**U**_**6**_**T**_**7**_G_8_T_9_T_10_A_11_C_12_ 3′	5′ L_3_ Motif R_1_ 3′	5′ G Motif 3′	5′ CG Motif G 3′
2AA	5′ T_1_G_2_A_3_A_4_**A**_**5**_**U**_**6**_**A**_**7**_G_8_T_9_T_10_A_11_C_12_ 3′	5′ L_3_ Motif R_2_ 3′	5′ G Motif 3′	5′ CG Motif G 3′
3AT	5′ C_1_T_2_G_3_C_4_**A**_**5**_**U**_**6**_**T**_**7**_A_8_A_9_T_10_G_11_C_12_ 3′	5′ L_1_ Motif R_1_ 3′	N/A	5′ CG Motif G 3′
4TA	5′ T_1_A_2_A_3_C_4_**T**_**5**_**U**_**6**_**A**_**7**_C_8_A_9_T_10_T_11_C_12_ 3′	5′ L_4_ Motif R_3_ 3′	N/A	5′ CG Motif G 3′

^a^All substrates pair adenine (NMR, simulations) or 2AP (fluorescence, kinetics) opposite uracil in the complementary strand. ^b^L_1_ = 5′ CTATATTGGAAGCT 3′, L_2_ = 5′ CTATGTTGGAAGCT 3′, L_3_ = 5′ CTAGAATGGAAGCT 3′, L_4_ = 5′ CTATATTCGCAGCT 3′, R_1_ = 5′ TCTGTACACGAAG 3′, R_2_ = 5′ TCAGTACACGAAG 3′, R_3_ = 5′ TCTCAACACGCTT 3′

### Sample preparation

Duplex DNA substrates used for the fluorescence-based experiments were prepared by annealing uracil-containing oligonucleotides with complementary strands containing 2AP opposite to the uracil. For the kinetic assays, a small excess of the 2AP-strand is preferable to an excess of the uracil-containing strand because the latter is also a substrate of UNG, and therefore its presence may affect the measured kinetic rates. For the kinetic assays, DNA substrates were prepared by annealing the strands at room temperature while monitoring the fluorescence intensity of 2AP in real-time. The uracil-containing strand was added to a known concentration of 2AP-containing strand, and the reduction in fluorescence intensity due to the formation of the duplex was measured until a small addition of the uracil strand did not result in a further decrease. The concentration of the resulting dsDNA substrate was calculated from the absorbance of the initial 2AP-containing strand and the volumes before and after adding the uracil strand. Traditional native polyacrylamide gel electrophoresis was used to confirm that the annealing procedure at room temperature was highly efficient and did not result in any measurable 2AP- or uracil-containing single strands. For the time-resolved and fluorescence quantum yield experiments, a slight excess of the U-strand is preferable to an excess of the 2AP-strand because 2AP in ssDNA is significantly brighter than 2AP in a duplex. Samples for these experiments were prepared as before and followed by the addition of a ~ 20% excess of the uracil-containing strand. In each case, we verified that further addition of the uracil-containing strand did not change the measured lifetimes or quantum yields. Duplexes for NMR experiments were prepared from complementary oligonucleotides mixed at 1:1 molar ratios that were heated to ~ 80 °C and annealed by cooling to room temperature for ~ 2 h.

### UNG kinetic assay and data analysis

**Figure 1:**
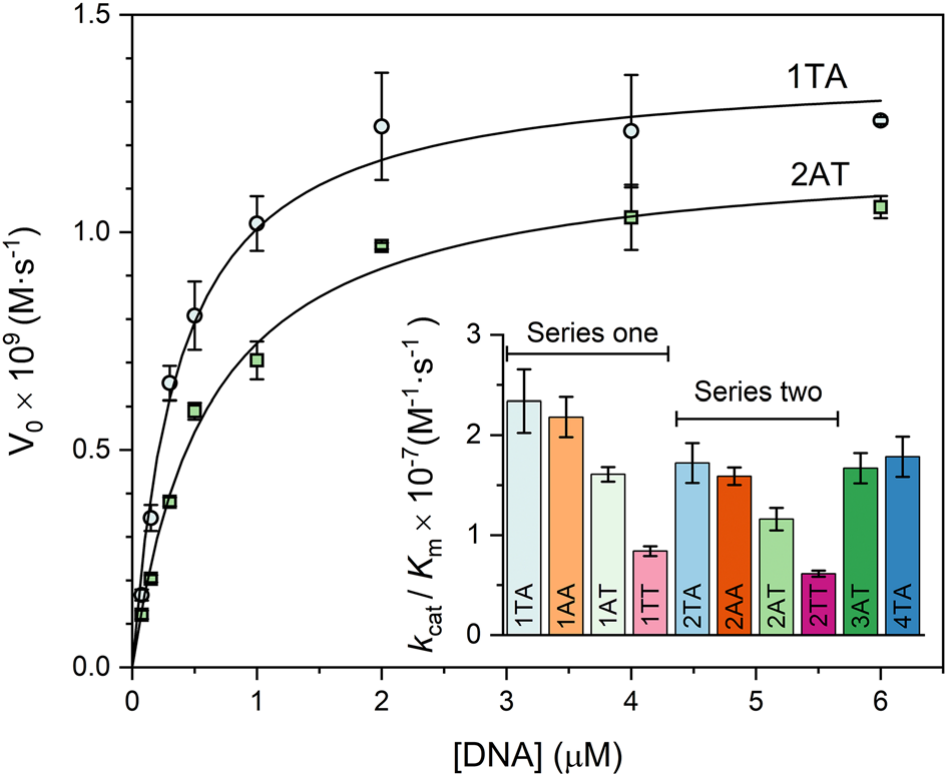
Experimental initial rates (V_0_) against substrate concentration for substrates 1TA (circles) and 2AT (squares). Results for all other samples are shown in Fig. S7. Each data point is the mean of at least three independent experiments. Error bars represent 95% confidence intervals. The data have been fitted to the Michaelis–Menten equation (see Table S4 for all Michalis-Menten parameters). Inset: Values of *k*_cat_/*K*_M_ for all substrates (see Table S4). Bar colors match the colors of the symbols used in other figures for the same sequences.

A continuous fluorescence kinetic assay was used to measure UNG activity on DNA substrates containing 2AP opposite to the uracil^24^. 2AP is highly fluorescent when exposed to water but is highly quenched in dsDNA. The cleavage of the dU glycosidic bond by UNG results in an aldehydic abasic site opposite to 2AP, and this environmental change leads to a large fluorescence increase. The increase in fluorescence intensity can be used to calculate the reaction rate in a continuous kinetic assay. The kinetic parameters measured in this way are indistinguishable from the values obtained using a traditional radioactivity-based electrophoresis assay with non-fluorescent substrates^24^. In addition, the perturbation introduced by the 2AP probe has been shown to have a negligible effect on both the measured dissociation and association kinetic constants of the UNG-DNA complex^25^. 600 μL of duplex DNA was placed in a quartz micro cuvette (optical path length 10 × 2 mm) and fluorescence intensity (λ_ex_ = 310 nm, λ_em_ = 370 nm) was monitored continuously before and after addition of UNG. Fluorescence intensities were corrected in real-time for potential fluctuations in the incident intensity over the long measurement times. A small amount of stock enzyme (5,000 U/mL) was diluted 40-fold in 1 × PBS buffer containing 1 mg/mL BSA, and this dilution was stored at 4 °C for no longer than six hours and used for all kinetic experiments performed the same day. For the kinetic experiments, 2 μL of this dilution were added to the cuvette containing the DNA substrate for a final concentration of UNG in the assay mixture of 0.16 nM. The initial velocity (V_0_, units of M·s^-1^) was obtained from the initial slope of the measured intensity (*F*(*t*)) as ${V}_{0}=\frac{{[S]}_{0}}{\Delta F}{\left(\frac{dF}{dt}\right)}_{0}$, where [S]_0_ is the concentration of DNA substrate (0.075–6 μM) and ΔF is the total change in fluorescence. A representative sample run and a sample calculation are shown in Fig. S1. Error bars in Figs. 1 and S7 represent 95% confidence intervals. To rule out systematic sources of error, for 1AT, 23 trials were performed at 0.3 μM concentration involving (1) at least four different purchased UNG stock solutions, (2) three different experimentalists, and (3) DNA substrates prepared from oligos purchased at different times. The V_0_ versus [DNA] curves were fitted using the Michaelis–Menten equation in Origin Pro (Northampton, MA) using the Lavenberg Marquardt iteration algorithm and using the reciprocal of the variances as weights.

### Time-resolved fluorescence

Time-resolved fluorescence intensity measurements were performed using the time-correlated single-photon counting (TCSPC) technique. A mode-locked Ti:Sapphire laser (Mira 900, Coherent) pumped by a frequency-doubled Nd:YVO4 laser (44% from an 18 W Verdi, Coherent) was used as the excitation source. The 130 fs light pulses (at 800 nm with a repetition rate of 250 kHz) were generated by a regeneratively amplified Ti:S laser system (RegA 9000, Coherent Laser). The pulses were sent to an optical parametric amplifier (OPA) to generate the excitation light at 620 nm and then frequency-doubled to obtain excitation pulses at 310 nm. Fluorescence emission was collected at a 90° geometry setting and detected using a double-grating monochromator (Oriel Instruments) and a microchannel plate photomultiplier tube (Hamamatsu R3809U-51). Decays were measured at three emission wavelengths (380, 390, and 400 nm) for global analysis as described below. The polarization of the emission was 54.7° relative to that of the excitation (magic angle). A single-photon counting card (Becker-Hickel, SPC-830) using two time windows (3.3 ns and 25 ns) was used for data acquisition. Instrumental response functions (IRF) were determined for both time resolutions. The typical IRF had a FWHM of 40 ps, measured from the scattering of Ludox sample at 310 nm. The three decays obtained at different emission wavelengths for each sample were fitted globally keeping the lifetimes as common parameters among the three data sets. This approach minimizes the problem of correlation between pre-exponential factors and lifetimes, which is common when fitting multi-exponential decays^26^. The fitting parameters were obtained through iterative reconvolution of the model function $F\left(\lambda ,t\right)={F}_{0}\left(\lambda \right)\sum_{i=1}^{n}{\alpha }_{i}\left(\lambda \right){e}^{-t/{\tau }_{i}}$ with the measured IRF using an in-home written software package (ASUFIT). Here, λ represents the emission wavelength, and $\sum_{i=1}^{n}{\alpha }_{i}\left(\lambda \right)=1.$ Lifetimes as long as ~ 8 ns and as short as ~ 30 ps are expected^26^, and in our experience, results are more robust and reproducible if the shorter components are determined from data measured with the highest time resolution achievable with the single-photon counting card (814 fs/channel, 2^12^ channels = 3.3 ns total), while the longer lifetimes are obtained from data measured with a wider time window. The decays measured using 3.3 ns acquisition windows were fitted using three exponential terms. A fourth term did not improve the quality of the fit. A new fit was then conducted using the decays measured with a 25 ns acquisition window (6.1 ps/channel) using four exponential terms but fixing the two shortest lifetimes (τ_1_ and τ_2_) to the values obtained in the previous fit. In this way, the fitting parameters in the second fit were τ_3,_ τ_4_ and ${\alpha }_{1-4}\left(\lambda \right).$ This procedure allows a more accurate determination of τ_1_ and τ_2_ (both below 0.5 ns) from measurements using 814 fs/channel resolution, and τ_3_ and τ_4_ (both over 1 ns) from measurements using 6.1 ps/channel resolution. Mean lifetimes were calculated for each sample as $\langle \tau \rangle = \sum_{i=1}^{4}{\alpha }_{i}{\tau }_{i}$ using the ${\alpha }_{i}$ values obtained in the second fit.

### Fluorescence quantum yields

Fluorescence quantum yields (ϕ) were determined relative to a reference as ${\phi }_{s}={\phi }_{R}\frac{{I}_{s}}{{I}_{R}}\times \frac{1-{10}^{-{A}_{R}}}{1-{10}^{-{A}_{S}}}$, where the subscripts “S” and “R” refer to the sample and reference, respectively, *I* is the integrated emission intensity measured over the entire fluorescence band, and A is the absorbance at the excitation wavelength (315 nm)^27^. Absorbances were kept below 0.05 to avoid inner filter effects. Experiments replacing the 2AP-containing strand with an adenine-containing strand were used to verify that the absorbance of the canonical bases at 315 nm was negligible compared to the absorbance measured with the 2AP-containing DNA. Steady-state emission fluorescence spectra were acquired on a PTI Quantamaster 4/2005SE spectrofluorimeter. Fluorescence spectra showed clear contributions from Raman scattering at 352 nm, and to account for these contributions a buffer sample was used as a blank and subtracted from the measurements containing 2AP-containing DNA. The free 2AP riboside is commonly used as a reference (${\phi }_{R}=0.68$ in water)^28^, but fluorescence intensities in duplex DNA are reduced 100-fold or more due to quenching, and therefore the determination of ϕ in DNA involves measuring very small fluorescence intensities. To improve accuracy, we performed five independent determinations of ϕ for the sample with the highest quantum yield (1AT) using free 2AP riboside in water as a reference. The average of five determinations was ϕ_1AT_ = 0.0103 (standard deviation = 6.5 × 10^–4^, 95% confidence interval = 8.1 × 10^–4^), and this value was subsequently used as a reference for the ϕ determinations of all other 2AP-DNA samples. Values listed in Table S1 are averages of 4 independent determinations. All standard deviations are 3% or lower.

### Fractional population of highly stacked species (α_0_)

The fractional population of dark (highly stacked) 2AP probes in the duplex DNA substrates was calculated from the sample mean lifetime ($\langle \tau \rangle$) and fluorescence quantum yield (ϕ) as ${\alpha }_{0}=1-\frac{{\tau }_{2AP}}{\langle \tau \rangle }\frac{\phi }{{\phi }_{2AP}}$, with ${\phi }_{2AP}=0.68$ and ${\tau }_{2AP}=10.2$ ns^28,29^. Errors were estimated as ${\Delta \alpha }_{0}={\left({\left(\frac{{\tau }_{2AP}}{{\phi }_{2AP}}\frac{1}{\langle \tau \rangle }\Delta\phi \right)}^{2}+{\left(\frac{{\tau }_{2AP}}{{\phi }_{2AP}}\frac{\phi }{{\langle \tau \rangle }^{2}}\Delta \langle \tau \rangle \right)}^{2}\right)}^{1/2}$. Values of $\Delta\phi$ were taken as the standard deviation of the quantum yield measurements for each sample. $\Delta \langle \tau \rangle$ values were estimated from the two repeats performed for each sample (Table S2). Because performing large numbers of repeats for individual samples in unfeasible due to time and cost, we calculated the percent deviation of each $\langle \tau \rangle$ determination (two values for each of the six measured samples) from the respective average for the same sample. We determined that, on average, $\langle \tau \rangle$ values are measured with a 2.5% precision. This value was then used to estimate $\Delta \langle \tau \rangle$ for each sample.

### NMR-detected imino proton exchange rate measurements

NMR samples were prepared in 3 mm tubes with DNA concentrations ranging from 2 to 4 mM with 5% v/v deuterium oxide. NMR experiments were performed on a Bruker 850 MHz Avance III HD spectrometer equipped with a 5 mm TCI CryoProbe, and a Bruker 600 MHz Avance III HD spectrometer equipped with a Prodigy probe. NMR spectra were processed and analyzed using Bruker TopSpin 4.1, MestReNova 14.2, and Matlab 2019b.

#### Resonance assignments

Two-dimensional ^1^H, ^1^H—Nuclear Overhauser Effect Spectroscopy (NOESY) experiments were recorded at 20 °C utilizing water suppression by excitation sculpting. The resulting 2D spectra were used to assign imino protons for each duplex using traditional methods (Figs. S2, S3)^30,31^. All assignments and subsequent experiments were collected at 20 °C.

#### Water inversion efficiency factor (E)

The water inversion efficiency factor was measured as previously described^32^, with a relaxation delay of 30 s, and is further described in the supplementary information (Fig. S4). Data processing and fitting were completed in Bruker TopSpin 4.1 and MestReNova 14.2.

#### Longitudinal relaxation rate of water (R_1w_)

The relaxation rate of water (*R*_*1w*_) was measured utilizing a previously described saturation-recovery method that is compatible with high Q cryoprobes^32^. Variable time delays ranged from 1 ms to 18 s. Data processing and fitting were completed in Bruker TopSpin 4.1 and Matlab 2019b for verification (Fig. S5). Determination of *R*_*1w*_ was completed using the TopSpin T1/T2 Module.

#### Imino proton longitudinal relaxation (R_1n_) and exchange rates (k_ex_)

The sum of the longitudinal imino proton relaxation rates (*R*_*1*_) and the imino proton solvent exchange rate (*k*_*ex*_) can be used to determine the longitudinal relaxation rate of each imino proton (*R*_*1n*_). A pseudo-two-dimensional experiment was implemented to determine the longitudinal relaxation of imino protons (*R*_*1n*_) and the imino proton exchange rates (*k*_*ex*_) following established methods^33^ utilizing a 24-point variable delay sequence ranging from 1 ms to 15 s. The respective spectra were processed in TopSpin 4.1, and the data were fit with MATLAB R2019b (MathWorks) using nonlinear least-squares fit, first fitting for the longitudinal relaxation of imino protons (*R*_*1n*_) followed by the exchange rate of imino protons (*k*_*ex*_). Representative fits of *R*_1n_ and *k*_*ex*_ are shown in Fig. S6. The *k*_*ex*_ was determined by fitting the individual peak areas to the equation:

1 $$\frac{A\left( t \right)}{{A_{0} }} = 1 - {{E \cdot k_{ex} } \mathord{\left/ {\vphantom {{E \cdot k_{ex} } {\left( {R_{1w} - R_{1n} } \right) \cdot \left( {e^{{ - R_{1n} \cdot t}} - e^{{ - R_{1w} \cdot t}} } \right)}}} \right. \kern-0pt} {\left( {R_{1w} - R_{1n} } \right) \cdot \left( {e^{{ - R_{1n} \cdot t}} - e^{{ - R_{1w} \cdot t}} } \right)}}$$

where *A(t)* is the area of the peak at exchange time point *t*, *A*_*0*_ is the peak intensity at equilibrium, *E* (determined experimentally) is the water inversion efficiency factor, *R*_*1w*_ (determined experimentally) is the water longitudinal relaxation rate, and *R*_*1n*_ (determined experimentally) is the sum of imino proton longitudinal relaxation rates and its solvent exchange rate. The reported errors were estimated from the fitting of *k*_*ex*_ (Table S3).

### Double mutant cycles and base-pair coupling analyses

The energetic impact of base substitutions was quantified from thermodynamic cycles that depict differences in transition state free energies: $\mathrm{\Delta \Delta }{G}_{1\to 2}^{\ddagger }=\Delta {G}_{2}^{\ddagger }-\Delta {G}_{1}^{\ddagger }$ , where indices 1 and 2 indicate two different DNA sequences^34^. Two cycles were constructed. The first was based on NMR data, where $\Delta {G}^{\ddagger }$ represents the barrier for imino exchange, and $\mathrm{\Delta \Delta }{G}_{1\to 2}^{\ddagger }=-RT \mathrm{ln}\left[\frac{{\left({k}_{ex}\right)}_{2}}{{\left({k}_{ex}\right)}_{1}}\right]$ . Differences between the 2TA, 2TT, 2AA, and 2AT sequences were assessed. The second cycle was based on *k*_cat_/K_M_ measurements, where $\Delta {G}^{\ddagger }$ represents the barrier for enzymatic uracil excision, and $\mathrm{\Delta \Delta }{G}_{1\to 2}^{\ddagger }=-RT \mathrm{ln}\left[\frac{{\left({k}_{cat}/{K}_{M}\right)}_{2}}{{\left({k}_{cat}/{K}_{M}\right)}_{1}}\right]$. In this cycle, differences between 2AT, 2TT, 2AA, 2TA, and 1AT, 1TT, 1AA, 1TA (see Table 1 for substrate nomenclature) were calculated. From these cycles, coupling free energies

2 $$\Delta G_{coup} = \Delta \Delta G_{AT \to TT}^{\ddag } - \Delta \Delta G_{AA \to TA}^{\ddag } = \Delta \Delta G_{AT \to AA}^{\ddag } - \Delta \Delta G_{TT \to TA}^{\ddag }$$

between the base pairs directly adjacent to uracil were assessed. ${\Delta G}_{coup}$ is zero when these base pairs are independent of each other, and nonzero when they are coupled and influence each other. Since the transition state free energies of the two cycles correspond to different processes, free energy differences and coupling free energies are expected to differ between the thermodynamic cycles.

### MD simulations

The dsDNA sequences of Table 1 were built in the unbent BII conformation using 3DNA^35^; U_6_ was base-paired to A_19_. Each strand was solvated in a rectangular TIP3P water box^36^ of 100 mM NaCl with a solvent layer of 15 Å in each direction. After energy minimizations, each system was heated from 100 to 300 K over 2.5 ns with a 1 kcal/(mol·Å^2^) harmonic restraint on all DNA atoms. During heating, flat bottom distance restraints with a force constant of 1 kcal/(mol·Å^2^) were added to the hydrogen bonds between the bases. After heating, the harmonic restraints on the DNA atoms were gradually removed over 1.2 ns, while restraints on the hydrogen bonds remained in effect. The latter were subsequently removed over an additional 3 ns. The unrestrained systems were then equilibrated for 400 ns, followed by at least 600 ns of production simulations. Heating and restraints removal were performed with Langevin dynamics in AMBER^37^, while the production runs were done with Langevin dynamics in OPENMM^38^. The simulations were performed in NPT, periodic boundary conditions were in effect, SHAKE^39^ was applied to all covalently bonded hydrogen atoms, and long-range electrostatic interactions were handled using the particle-mesh Ewald method^40^. All simulations used the AMBER OL15 DNA force field^41^; deoxyribose parameters for U were taken from T deoxyribose. Convergence was assessed by monitoring cumulative averages of DNA bending and total winding angles. In addition, all trajectories were decorrelated using pymbar^42^, and all properties were calculated from 100 decorrelated frames per trajectory. If needed, simulations were extended for additional blocks of 500 ns until convergence. Simulations were run in triplicate for each strand.

Geometric analyses of the DNA base and step parameters were performed with 3DNA^35^. DNA bending angles (*ɸ*) were calculated from tilt, roll, and twist base step angles using the MADBEND procedure^43,44^. Bending persistence lengths (BPLs) were calculated from: $BPL = - L\frac{{\partial {\text{ln}}P\left(\upphi \right)}}{{\partial \left( {1 - \cos \left(\upphi \right)} \right)}}$^45^, where *L* is the contour length, and *P*(*ɸ*) the probability of observing a particular bending angle. Contour lengths were calculated from the sum of the helical rise; to account for fraying, the terminal base steps were excluded from the *ɸ* and BPL analyses. Torsional persistence lengths (TPLs) were calculated from the variance of the total winding angle (${\Delta }_{\Omega }^{2}$): $TPL=L/{\Delta }_{\Omega }^{2}$^46^. The total winding angle was calculated as the sum of the individual twist steps for each sequence. To account for fraying and base flipping, the two terminal base steps and those neighboring U_6_ were excluded from the sum.

Extrahelical flipping of uracil was assessed by monitoring the hydrogen bonding distance between HN_3_ of U_6_ and N_1_ of its complementary A_19_, and the flipping angle. This flipping angle was taken as the pseudo-dihedral angle between the center of mass (COM) of the base ring of U_6_, the COM of the U_6_ backbone, the COM of the backbone of residue 8, and the COM of the backbone of the base complementary to U_6_^45,45^. Based on visual inspections of the trajectories, U_6_ was considered flipped out when the U_6_ − A_19_ hydrogen bond distance exceeded 4.5 Å and the pseudo-dihedral angle was greater than 40 or less than − 40°. Negative pseudo-dihedral angles correspond to major groove flipping, while positive angles correspond to minor groove flipping.

## Results

### DNA substrates

Relative excision efficiencies for uracils embedded within a long (> 6 kbp) viral dsDNA have previously been reported^5^. Inspection of those results suggests that AUT sequences are generally poorer UNG substrates than TUA sequences despite similar overall AT/GC content. Based on these published data, we designed dsDNA substrates containing uracil in TUA or AUT contexts, and adenine opposite uracil (Table 1). An initial set of substrates (1TA, 2AT, 3AT and 4TA) were designed to span a wide range of uracil excision efficiencies. For uracils embedded in a viral dsDNA genome, substrates 1TA and 4TA represent the high and low end of removal efficiencies in TUA contexts (100% and 50%, respectively), while substrates 3AT and 2AT represent the high and low end in AUT contexts (35% and 10%, respectively)^5^. Substrates 1AT and 2TA were then designed to evaluate the impact of swapping the flanking A and T bases of the substrates with the best (1TA) and worst (2AT) removal efficiency in the original set. Lastly, substrates containing uracil in a AUA (1AA and 2AA) or a TUT (1TT and 2TT) context were designed to evaluate the contributions of the uracil-flanking nucleotides.

### Kinetics of uracil removal by UNG

UNG activity was measured as described in Materials and Methods; an example is given in Fig. S1. Although 2AP introduces slight local conformational perturbations^48,49^, its effects on enzyme binding and kinetics are marginal^25,26,50,51^. Calculated Michaelis–Menten parameters (*K*_M_ and *k*_cat_, Figs. 1 and S7) and specificity constants (*k*_*cat*_/*K*_M_) are listed in Table S4. *K*_M_ values fall within the range of previous measurements^23,52–54^. For substrates 1TA, 2AT, 3AT, and 4TA, *k*_*cat*_/*K*_M_ ratios parallel the percent uracil removal efficiencies reported previously^5^ in longer and more complex viral DNA: 1TA > 4TA > 3AT > 2 AT (Fig. 1, inset). For substrates 1AT and 2TA, whose core motifs are identical to 1TA and 2AT except for the swapped flanking bases, *k*_*cat*_/*K*_M_ ratios are higher in TUA than AUT contexts (i.e. 1TA > 1AT and 2TA > 2AT, Fig. 1 and Table S4). Competition experiments (Supporting Information) using UNG acting on two different uracil-containing substrates present in the same reaction mixture confirm that UNG’s specificity is greater in TUA than AUT contexts, and that substrate specificity is not solely determined by the flanking bases (see also Fig. 1, inset).

### Fluorescence quantum yields and time-resolved fluorescence

Unlike the canonical DNA bases, 2-aminopurine (2AP) is highly fluorescent when free in solution and strongly quenched when incorporated into DNA^26^. Inter-base interactions with its neighboring bases in duplex DNA give rise to a multiexponential decay that reflects the highly heterogeneous environment sensed by the probe. We used steady-state and time-resolved 2AP fluorescence to probe DNA dynamics around the uracil lesion. Consistent with previous reports^55–58^, four exponential terms with lifetimes ranging from tens of picoseconds to nanoseconds were needed to fit the time-resolved TCSPC data (Table S2):

3 $$I\left( t \right) = I_{0} \sum\nolimits_{i = 1}^{4} {\alpha_{i} e^{{ - t/\tau_{i} }} } ,\quad \sum\nolimits_{i = 1}^{4} {\alpha_{i} = 1}$$

**Figure 2:**
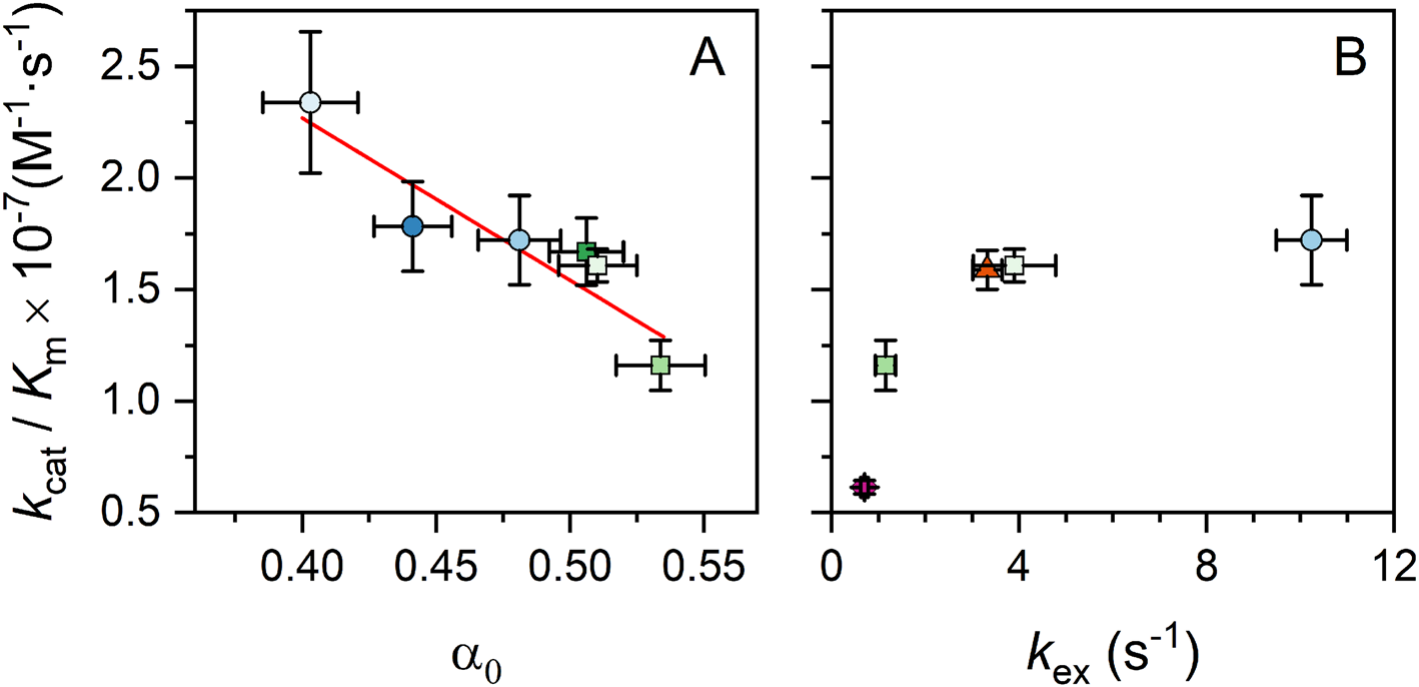
(**A**) Inverse correlation between the measured specificity constants (*k*_cat_/*K*_M_) and α_0_, a fluorescence-derived experimental observable that measures the degree of base stacking in the region surrounding the uracil. (**B**) Correlation between *k*_cat_/*K*_M_ and the NMR-measured exchange rate constants (*k*_ex_) for the uracil imino proton.

Lifetimes in the picosecond timescale have been reported for 2AP in dsDNA using ultrafast methods^59^, indicating that a fraction of the emitting 2AP fluorophores has lifetimes shorter than the TCSPC resolution (~ 40 ps). The fractional population of the highly stacked probes that give rise to these ultrafast lifetimes (denoted by α_0_) was determined from the average lifetimes (Table S2) and the measured fluorescence quantum yields (Table S1)^29^. Results are shown in Table S5 and Fig. 2.

Seibert et al. reported fluorescence quantum yields and lifetimes of 2AP opposite a uracil in two different 19 bp DNA substrates: one containing uracil flanked by two As (AUA, high UNG efficiency) and one containing uracil flanked by two Gs (GUG, low UNG efficiency)^23^. The AUA sequence had a shorter average lifetime (0.32 ns) than GUG (2.48 ns), which was interpreted as AUA being more flexible and therefore leading to more efficient dynamic quenching. Guanine, however, is an efficient quencher of 2AP, and 2AP lifetimes as low as 400 fs, were measured for 2AP in the vicinity of G^59^. The surprisingly long average lifetime reported for GUG (2.48 ns), therefore, likely reflects the fact that most of the 2AP population is quenched dynamically with lifetimes shorter than the resolution of the experiment (the shorter lifetimes reported were ~ 200 ps while the typical resolution of TCSPC measurements is about 40 ps). Had the authors measured the short lifetimes with high relative amplitudes expected for 2AP in the vicinity of G, the calculated 〈τ❭ would have been significantly shorter and likely below the value measured for AUA. These arguments illustrate the problems with interpreting relative 2AP average lifetimes in terms of substrate flexibility. Because measured average lifetimes are sensitive to instrumental resolution, we favor the currently accepted view that the relative contributions of each lifetime (α_i_), but not the lifetimes themselves, are useful measures of the degree of base stacking, and therefore reflect substrate flexibility^26^.

The excited 2AP population is expected to be partitioned between several different local environments that lead to different quenching efficiencies. The normalized amplitudes in Eq. (3) (α_i_ ), measure the fractional population of each conformation detectable by TCSPC, from more stacked (α_1_), to more exposed to the solvated environment (α_4_)^26^. Here, we focus on the remaining population of 2AP molecules that cannot be detected by TCSPC (α_0_). This population emits with lifetimes shorter than the resolution of the experiment (~ 40 ps) due to ultrafast quenching. All calculated α_0_ values are quite high (α_0_ > 0.4 for all sequences, Table S5), but values are higher for substrates containing uracil in a AUT context compared to TUA. A higher α_0_ value indicates a higher fraction of highly stacked 2AP fluorophores, which we interpret as an indication of a less deformable substrate.

Substrates 1AT and 2TA were designed from parent substrates with high (1TA) and low (2AT) UNG efficiency to test the hypothesis that differences in substrate deformability determine preferential repair efficiencies. Swapping the flanking A and T while keeping all other bases constant affects stacking interactions in the vicinity of the uracil without changing the melting temperature. This swap resulted in a higher fraction of highly stacked 2AP probes for substrates with AUT contexts, i.e. α_0_ (1AT) > α_0_ (1TA) and α_0_ (2AT) > α_0_ (2TA). As noted above, *k*_cat_/*K*_M_ values follow the opposite trend, suggesting a correlation between substrate deformability and uracil removal efficiency by UNG. A strongly negative correlation between k_cat_/*K*_M_ and α_0_ was indeed observed for all sequences (Fig. 2A), indicating that more flexible sequences have higher repair rates.

### Molecular Dynamics (MD) Simulations

**Figure 3:**
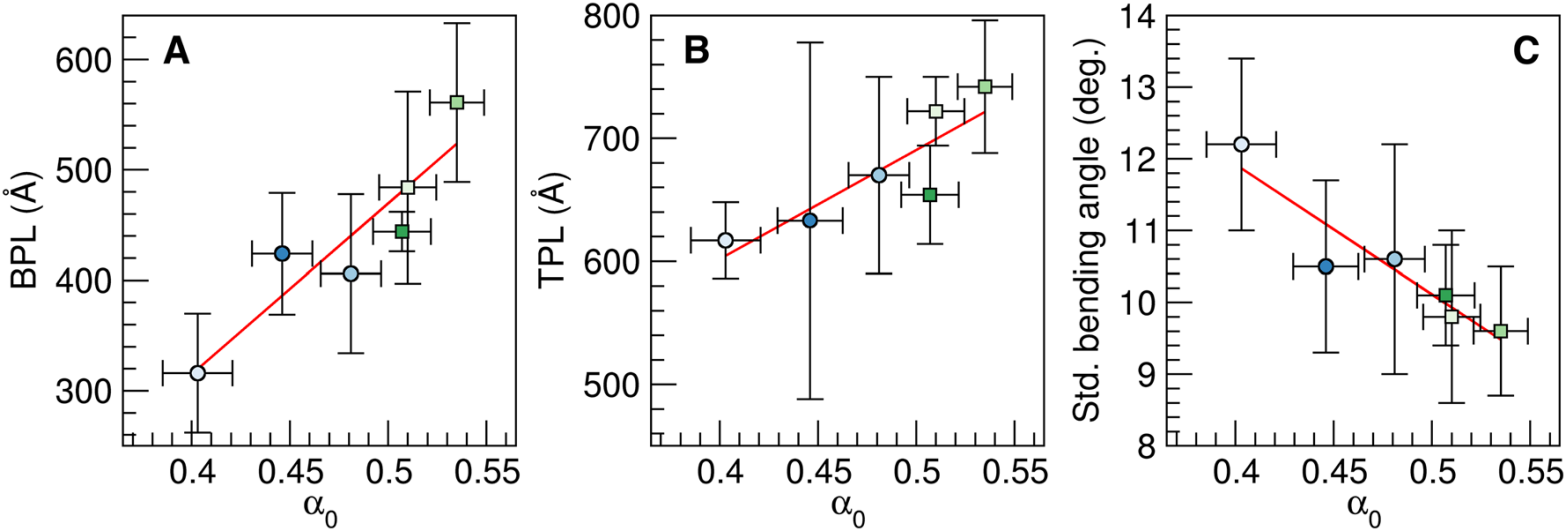
Correlation of MD properties with α_0_. (**A**) Bending persistence length (in Å), (**B**) torsional persistence length (in Å), (**C**) standard deviation of the bending angle (in degrees). Averages and standard deviations over 3 MD replicas. Red lines show linear regression of the data; correlation coefficients are 0.981 (**A**), 0.869 (**B**), and − 0.939 (**C**).

The flexibility of the various DNA duplexes (Table 1) was quantified by MD. Calculated bending persistence lengths showed that all TA sequences and 3AT were more flexible than undamaged DNA (which has a persistence length of ~ 500 Ȧ)^60,61^, while the 1AT, 2AT, 1TT, 2TT, 1AA, and 2AA sequences were similar to undamaged DNA in bending rigidity (Table S6). We observed a clear distinction between the TA and AT sequences, with the former having lower bending persistence lengths than the latter. The AA and TT sequences were least bendable. The higher flexibility of the TA sequences was largely echoed in calculated torsional persistence lengths: the AT sequences generally had larger torsional persistence lengths than the TA sequences. The only exception was the 3AT sequence, which was torsionally more flexible than the stiffest TA sequence (2TA). Torsional persistence lengths of the AA and TT sequences were similar to the AT sequences. The standard deviation of the bending angle was highest for the TA sequences, indicating large bending flexibility of these sequences, and lowest for AA/TT, indicating higher rigidity (Table S6). Overall, calculated properties indicated the highest flexibility for the TA sequences and the lowest flexibility for the AA and TT sequences. Figure 3 shows that these three properties are strongly correlated with the α_0_ values obtained from fluorescence measurements. The correlation coefficient (*r*) was 0.918 for the bending persistence length, 0.869 for the torsional persistence length, and − 0.939 for the standard deviation of the bending angle. Moreover, ranking of the sequences by flexibility was largely similar for these MD measures and α_0_.

The sequences displayed markedly different local dynamics around the lesion. Figure S8 shows the shift, slide, and rise translational step parameters, and tilt, roll, and twist rotational parameters for the central A_5_U_6_A_7_, A_5_U_6_T_7_, T_5_U_6_A_7_, and T_5_U_6_T_7_ steps. These values were averaged over all trajectories and all sequences; reported standard deviations are a measure of the base step flexibilities. Interestingly, differences in UA and AU step flexibilities depend on context and do not completely mirror the behavior of TA and AT steps of undamaged DNA^18,19,21^. For example, UA is particularly flexible in the TUA motif, but rigid in AUA. The TUA motif was by far most flexible, displaying large flexibilities in all step parameters of both steps. Some asymmetries in the UA and TU steps of this motif were observed. The roll angle of its UA step was more flexible than its TU step, its TU step was more flexible than UA in slide and twist, while both steps had similar flexibilities for the other parameters. Second most flexible was AUT. Its UT step was more flexible in roll, twist, shift, and rise, its AU step was more flexible in slide, and its tilt flexibility was similar for both steps. In contrast, all steps of the AUA and TUT motifs displayed low flexibilities.

The main reason for the high flexibility of the central base steps of the TUA sequences was extra-helical base flipping of U_6_ (Table 2), which was observed in all TUA sequences. Flipping was reversible and would occur throughout the simulations, but in 2TA and 4TA U_6_ remained extra-helical for nearly the entirety of the simulations. Base flipping was also observed in the AUT sequences, particularly in the 3AT and 1AT sequences, but this was by far not as prominent as when uracil was in a TUA context. 1AA and 2AA displayed even less flipping than the AUT sequences, and flipping did not occur in 1TT and 2TT.

**Table 2:** Average and standard deviation of the time U6 is intra-helical over three MD replicas.

Sequence	% Time U_6_ is intra-helical
2TA	0.9 ± 0.9
4TA	6.3 ± 6.6
1TA	15 ± 17
3AT	57 ± 35
1AT	71 ± 28
2AT	96.3 ± 1.9
1AA	96.2 ± 1.0
2AA	98.9 ± 0.4
1TT	100.0 ± 0.0
2TT	100.0 ± 0.0

**Figure 4:**
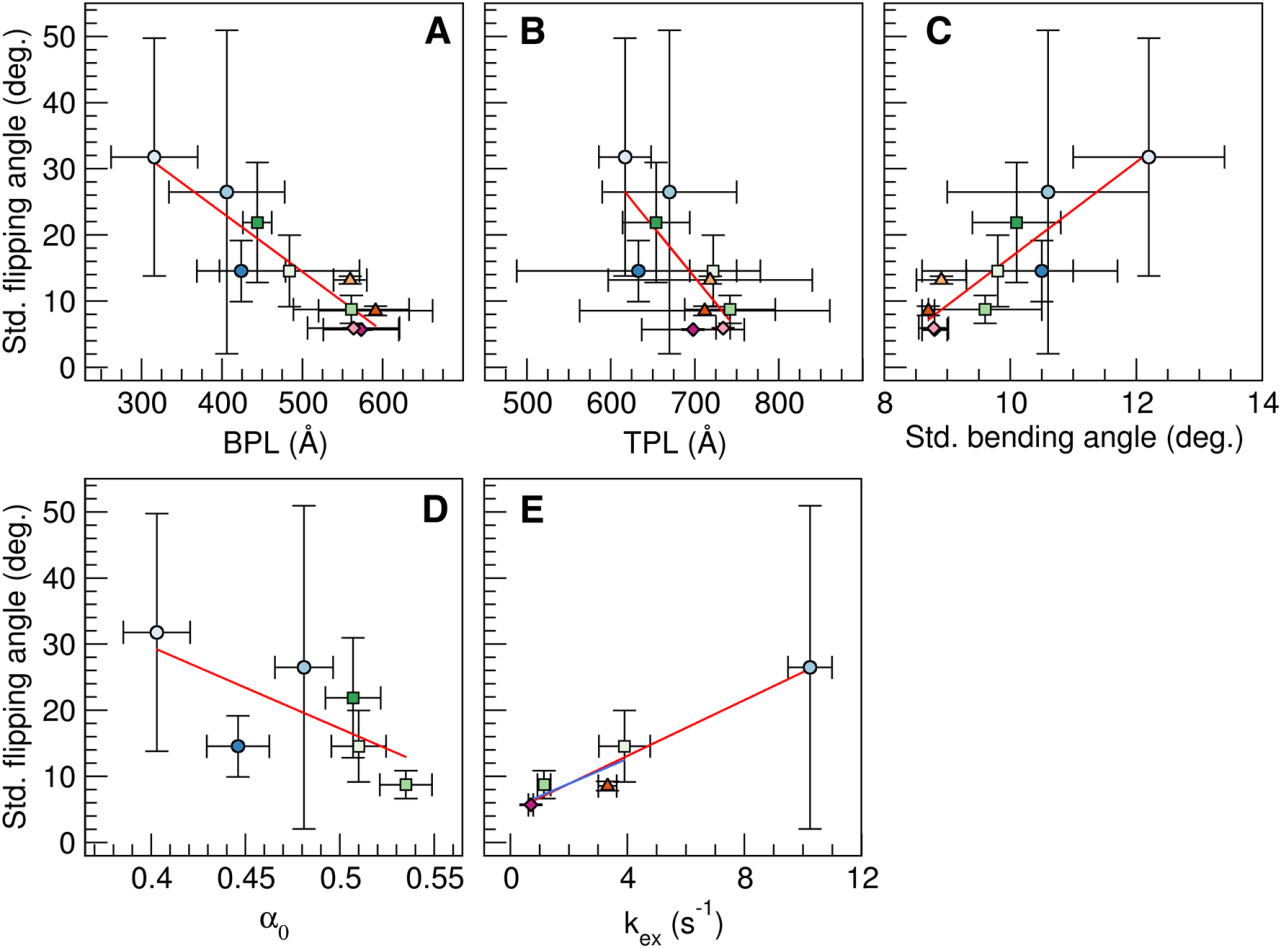
Correlation of the standard deviation of the flipping angle with the bending (**A**) and torsional persistence length (**B**), the standard deviation of the bending angle (**C**), α_0_ (**D**) and *k*_ex_ of U_6_ (**E**). Averages and standard deviations over 3 MD replicas. Red lines show linear regression of all data with correlation coefficients of − 0.972 (**A**), − 0.763 (**B**), 0.897 (**C**), − 0.694 (**D**), and 0.972 (**E**), blue line the regression excluding 2TA with a correlation coefficient of 0.799 (E).

Flipping of U_6_ was highly correlated to bending motions. The standard deviation of the flipping angle was highly correlated to the bending persistence length and the standard deviation of the bending angle, with correlation coefficients of -0.972 and 0.897, respectively (Fig. 4). The correlation to the torsional motion was weaker, with a correlation coefficient of − 0.763 for the torsional persistence length. Flipping would start toward the major groove (negative flipping angles). The fully flipped U would either remain in the major groove or start interacting with the minor groove, thereby changing the flipping angle to positive values. Since flipping was prevalent in the TA sequences, this behavior led to large standard deviations over the TA replicas (Fig. 4).

### NMR-detected imino proton exchange rates

**Figure 5:**
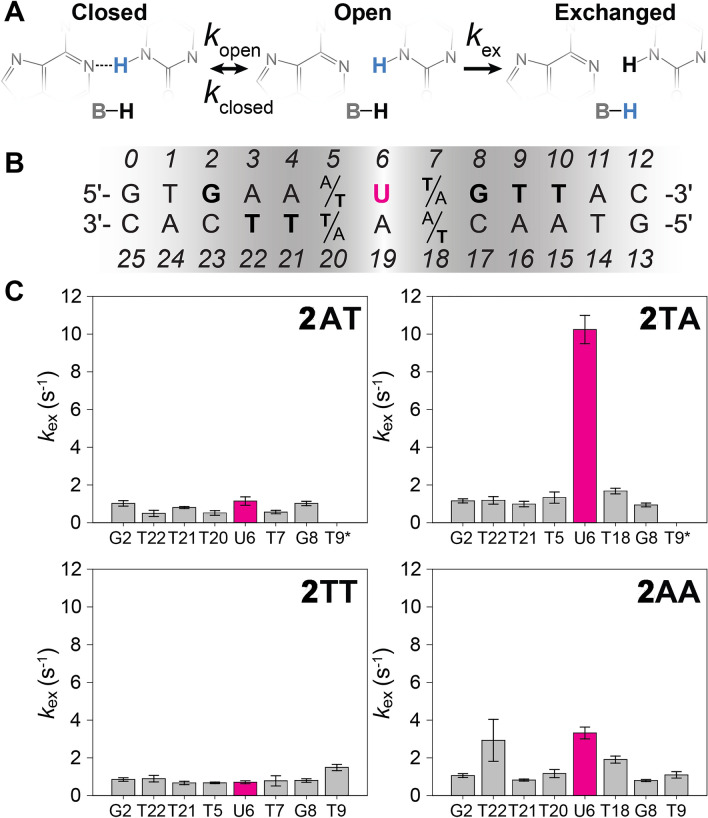
Imino proton exchange rates (*k*_ex_) of individual base pairs in each of the two-series substrates in isolation confirm MD and fluorescence dynamics studies. (**A**) Schematic representation of the two-state model for base pair opening and imino exchange kinetics in nucleic acids. When the *k*_ex_ is not the rate-limiting step, the *k*_ex_ is directly proportional to the stability of the base pair and provides insight into DNA duplex dynamics. (**B**) Series two UNG substrate sequences, with the central uracil (U_6_) highlighted in magenta. (**C**) Imino proton exchange rates (*k*_ex_) of individual base pairs in 2AT, 2TA, 2TT, and 2AA substrates. The uracil exchange rate corresponds to substrate efficiency. Error bars represent the propagated fitting error.

We probed individual base pair imino proton exchange rates of UNG substrates with solution NMR spectroscopy. These rates are directly related to nucleic acid breathing motions, whereby the imino base pair protons in transiently open states can exchange with water (Fig. 5A)^33,62,63^. The imino proton exchange rate (*k*_*ex*_) provides insight into the base pair stability and duplex dynamics at the single base-pair level. As indicated by the MD simulations, we hypothesized that the *k*_*ex*_ of the target uracil (U_6_, Fig. 5B) will be sequence-dependent, and that a higher *k*_*ex*_ for central base pairs reflects more suitable UNG substrates. The imino protons for each NMR duplex listed in Table 1 were assigned (Fig. S3), and individual base pair *k*_*ex*_ values were measured. The 1TA exchange rates were not characterized due to significant resonance overlap of uracil (U_6_) with T_18_ (Fig. S3) which impeded accurate deconvolution and analysis of the U_6_ and T_18_ resonances in *R*_1n_ and *k*_*ex*_ experiments. The UNG substrates from series one were therefore not included in the NMR analyses. The NMR data used for fitting the *k*_*ex*_ are deposited under BMRB Entry ID 51612.

Imino proton NMR spectra are only observed in the presence of base-pairing^64,65^; due to fraying, terminal duplex base-pairs were therefore not observed. Though most of the surrounding exchange rates were comparable between sequences, the central uracil exhibited strikingly distinct *k*_*ex*_ rates that varied by more than ten-fold across sequences (Fig. 5C). Differences in uracil imino exchange rates demonstrate a strong dependence on adjacent base pairs. In 2AT and 2TA, the *k*_*ex*_ significantly increases as the result of a conserved change in sequence order of the surrounding base pairs. Given the significant differences in dynamics observed by fluorescence, MD simulations, and NMR when swapping between AT and TA bases flanking the central uracil, we measured imino proton exchange rates for 2TT and 2AA to elucidate whether the 5′ T or 3′ A relative to uracil dictates substrate flexibility and UNG efficiency. Interestingly, the sequences with the highest uracil imino exchange rates are those with adenine 3′ to U_6_ (A_7_). The 2TA duplex measures the highest U_6_ *k*_*ex*_ of 10.24 ± 0.75 s^−1^ while 2AA is intermediate with a *k*_*ex*_ of 3.32 ± 0.31 s^−1^ (Fig. 5C). Our experiments identify that the base pair at the 3′ side of uracil is most influential on the *k*_*ex*_ of U_6_, demonstrated by the nearly 15-fold difference in *k*_*ex*_ between 2TA and 2TT (10.24 ± 0.75 s^−1^ and 0.70 ± 0.08 s^−1^, respectively). This could suggest a structural and/or energetic hindrance to UNG efficiency in repairing such sequences. A positive correlation between *k*_*ex*_ of U_6_ and k_cat_/*K*_M_ was observed (Fig. 2B), indicating that more flexible sequences have higher repair efficiencies.

### Thermodynamic cycle analysis

**Figure 6:**
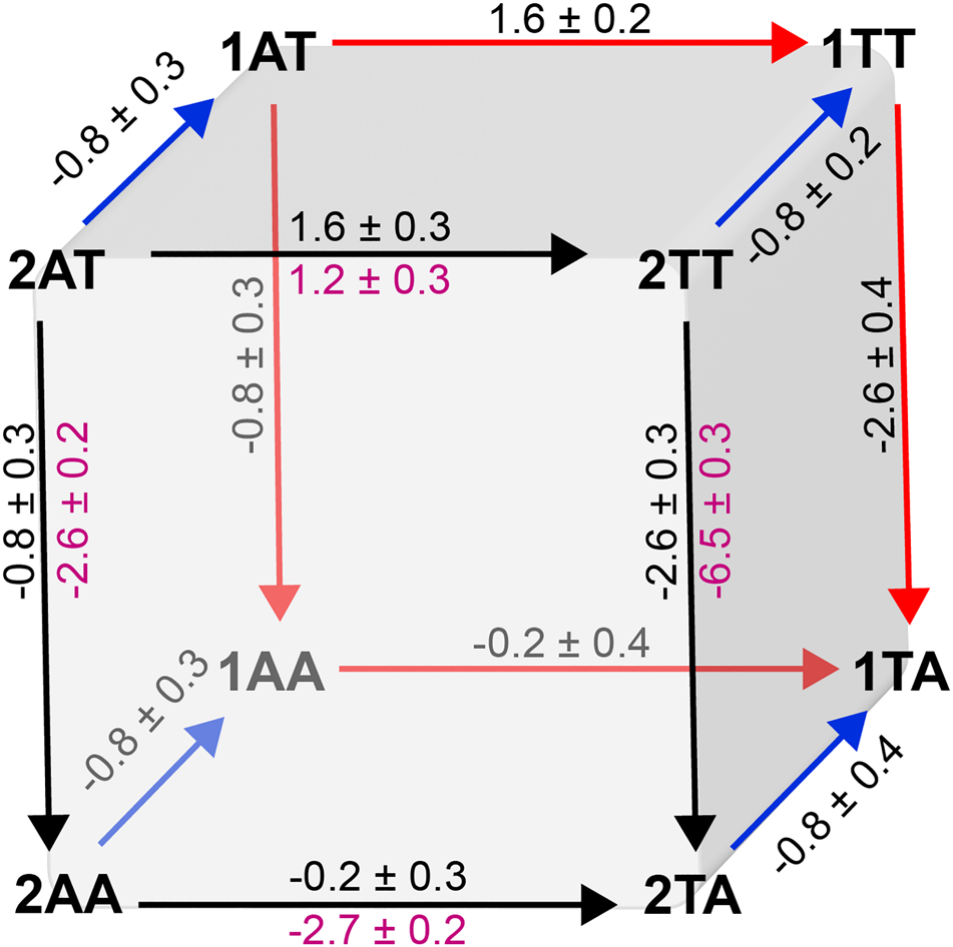
Double mutant cycle analysis for substrate series one (back face, red arrows) and substrate series two (front face, black arrows). All values represent $\Delta \Delta {G}_{1\to 2}^{\ddagger }$ in kJ/mol. Black values for substrate series one and two were calculated from *k*_cat_/K_m_ values of Table S4, magenta values for substrate series two from *k*_ex_ values of U_6_ as reported Fig. 5.

Given that the base identity 3′ to uracil most significantly contributes to substrate flexibility, we probed allosteric coupling between the 5′ and 3′ uracil flanking positions. Thermodynamic cycle analysis of the NMR data ($${k}_{ex})$$ measured for substrate series two identifies that there is coupling between these positions with a ΔG_coup_ of 3.9 ± 0.4 kJ/mol (Eq. 2 and Fig. 6). Based on the finding that substrate dynamics govern UNG activity, we expected coupling between the 5′ and 3′ flanking uracil positions in enzyme kinetics as well. This was validated by a thermodynamic cycle of substrate series two using $$\Delta {G}^{\ddagger }$$ values from *k*_cat_/*K*_M_ measurements, yielding a coupling energy of 1.8 ± 0.6 kJ/mol (Fig. 6). Thermodynamic theory dictates that this allosteric coupling should be preserved independent of the surrounding sequence, and to confirm this, a thermodynamic cycle was carried out with substrate series one. As expected, we obtained the same coupling energies in both cases (horizontal lines in Fig. 6).

## Discussion

The first uracil DNA glycosylase (UNG) was identified almost 50 years ago in *E. coli*^66^, and since, has been found across mammals, plants, bacteria, and viruses. While UNG is well characterized functionally and structurally, the observation that DNA substrate sequence significantly impacts its enzymatic efficiency has not been fully explained. To deconvolute the physical DNA substrate properties that impact UNG function, we employed a set of dsDNA UNG substrates that encompasses variable enzymatic efficiencies. UNG activity was correlated to substrate uracil dynamics by fluorescence, NMR, and MD. These studies identified both proximal and distal substrate features whose contributions impact UNG activity.

Uracil recognition by human and *E. coli* UNG relies on trapping extrahelical uracils that are spontaneously exposed by thermally induced base pair opening motions^12,13^. NMR imino proton exchange measurements have shown that U:A base pairs open with rate constants that are about one order of magnitude faster than T:A base pairs in the same sequence context^14^, and this difference has been proposed to contribute to the mechanism by which UNG discriminates between thymine and uracil. Here, we report uracil imino exchange rates that vary nearly 15-fold across sequences. These results support the notion that the sequence context surrounding the uracil modulate its opening dynamics and ultimately regulate the rate of uracil removal. To establish a correlation between substrate flexibility and UNG activity, we determined the UNG Michaelis–Menten constants for ten designed DNA substrates containing uracil flanked by either A or T bases (Figs. 1, S7 and Table S4). This experimental design allowed us to focus on the effects of substrate flexibility without affecting the melting temperature and stability of the duplex. UNG specificity constants (*k*_cat_/*K*_M_) were calculated and used to evaluate relative UNG substrate preferences. We identify that the UNG specificity constant (*k*_cat_/*K*_M_) is generally smaller for substrates containing a thymine 3′ to the uracil. Replacing the 3′ thymine in substrates 1TT and 2TT with an adenine (TUT → TUA), results in a *c.a.* threefold increase in *k*_cat_/*K*_M_. Swapping A and T around the uracil in substrates 1AT and 2TA (AUT → TUA) also results in an increase in *k*_cat_/*K*_M_.

The fact that UNG recognizes spontaneously exposed uracil suggests that sequence effects in *k*_cat_/*K*_M_ are due to inherent differences in the flexibility of the DNA helix around the uracil. Accordingly, we observe an inverse correlation between *k*_cat_/*K*_M_ and α_0_, a fluorescence-derived observable that measures the degree of base stacking around the uracil (Fig. 2A). Values of α_0_ are smaller for TUA sequences than for AUT, indicating that uracil is less dynamic in the second group. Similarly, a thymine 3′ to U results in lower exchange rate constants for the uracil imino proton (Table S3 and Fig. 2B), and we observed a correlation between *k*_cat_/*K*_M_ and *k*_ex_ for the five substrates for which we were able to obtain *k*_ex_ constants by NMR (Fig. 2B). This further supports the notion that *k*_cat_/*K*_M_ is greater for substrates containing the uracil in more flexible contexts. We note that there is no clear correlation between *k*_cat_ and α_0_ (Fig. S9), indicating that the values of *K*_M_, but not *k*_cat_, are dictated by the mechanical properties of the substrate. This is consistent with current mechanistic knowledge that UNG *k*_*cat*_ is limited by product release^67,68^.

MD data suggests that the fluorescence spectroscopy-derived α_0_ values and the NMR-determined U_6_-*k*_ex_ values measure different aspects of substrate motion. Values of α_0_ correlate more strongly with DNA bending than uracil flipping (Figs. 3, 4), while U_6_-*k*_ex_ values correlate more strongly to base flipping than DNA bending (Figs. 4, S10). Nevertheless, these motions are coupled (Fig. 4), and given the correlation of α_0_ and U_6_−*k*_ex_ with *k*_cat_/*K*_M_ (Fig. 2), both motions contribute to UNG substrate recognition and uracil excision. The necessity of both motions is consistent with crystal structures, in which DNA in complex with UNG is both flipped and bent (by an average of 33°)^10,13,69–73^. The stronger correlation of α_0_ with *k*_cat_/*K*_M_ (Fig. 2) is intriguing since it would indicate that excision is more strongly correlated to DNA bending than base flipping. The observation that *K*_M_, but not *k*_cat_, is governed by the mechanical properties of the substrate, indicates that DNA bending favors the formation of the enzyme–substrate complex. Our MD data shows that DNA bending correlates with base flipping, which is consistent with previous studies that show that DNA bending facilitates base flipping by pushing the system up in energy^74,75^.

For a given substrate, changing one of the bases flanking the uracil affects *k*_cat_/*K*_*M*_ in a way that depends on the identity of the other. For example, for substrate series one (i.e., 1AT, 1AA, 1TT, and 1TA) and two (i.e., 2AT, 2AA, 2TT, and 2TA), the effect of replacing an adenine 3′ to uracil with thymine is much greater when the base 5′ to uracil is T than A. Similarly, the effect of substituting an adenine 5′ to uracil by thymine is much greater when the base 3′ to uracil is T than A. The thermodynamic coupling between the bases flanking the uracil was analyzed by two types of double mutant cycles, one using data from solution NMR on the isolated substrates, and one from UNG activity (Fig. 6)^34^. It is evident from both cycles that $\mathrm{\Delta \Delta }{G}_{AT\to AA}^{\ddagger } \ne \mathrm{\Delta \Delta }{G}_{TT\to TA}^{\ddagger }$ and $\mathrm{\Delta \Delta }{G}_{AT\to TT}^{\ddagger } \ne \mathrm{\Delta \Delta }{G}_{AA\to TA}^{\ddagger }$, indicating that substituting one of the bases adjacent to uracil affects the energy of the transition state in a manner that depends on the identity of the other one. In other words, the sites directly 5′ and 3′ of uracil are allosterically coupled. Substituting a thymine 3′ to uracil by adenine (vertical edges of the cube in Fig. 6) stabilizes the transition states, but in both cycles, effects are more significant when the base 5′ to uracil is thymine. Similarly, substituting a thymine 5′ to uracil by adenine stabilizes the transition state when the base 3′ to uracil is thymine, but destabilizes it when adenine is in this position. This allosteric coupling is quantified by the coupling energy, with a value of 3.9 ± 0.4 kJ/mol for the *k*_ex_-based cycle with substrate series two, and values of 1.8 ± 0.6 kJ/mol and 1.8 ± 0.5 kJ/mol for the *k*_cat_/*K*_M_ -based cycle with substrate series two and one, respectively. While magnitudes of coupling necessarily differ between the two cycles, the coupling has the same sign in both cases, which supports the conclusion that UNG catalysis correlates with substrate dynamics. Coupling energies are higher for the imino proton exchange-based cycle compared to the *k*_cat_/*K*_M_-based cycle. Considering that the former measures coupling in the isolated substrates, these results point to the role of the enzyme in reducing the coupling energy, consistent with the fact that UNG must be capable of removing uracil in diverse sequence contexts.

For the *k*_cat_/*K*_M_-based cycle, coupling energies for substrate series one and two are identical, within error, indicating that the effect of a single change in a base adjacent to uracil is independent of the identity of the bases that differ between substrate series one from series two (i.e., $\Delta \Delta {G}_{1XY\to 1XX}^{\ddagger }=\Delta \Delta {G}_{2XY\to 2XX}^{\ddagger }$, where X and Y are A or T). This indicates that all the combined differences between substrates one and two have a constant effect in *k*_cat_/*K*_M_ that is independent of the identity of the bases flanking the uracil. Consistent with this, all values of $\Delta {G}_{2XY\to 1XY}^{\ddagger }$ are the same for any combination of X and Y (lines connecting the thermodynamic squares of substrates 1 and 2 in Fig. 6). Although results show that sequence effects are not limited to the bases immediately surrounding the uracil lesion (*k*_cat_/*K*_M_ values are 1.4-fold greater for substrate series one), for these sequences, effects appear to be additive to the effects of the bases flanking the uracil. Based on this analysis, we conclude that the bases adjacent to the uracil have the greatest impact in both substrate flexibility and UNG activity, and that the bases not immediately surrounding the uracil provide a secondary level of modulation.

While our study focused on *E. coli* UNG, we anticipate the results to be broadly applicable because the catalytic cores of different UNGs are closely related. For example, the root-mean-square deviation between all Cα positions of human and *E. coli* UNG enzymes is just 0.9 Å^76,77^. UNGs are also structurally similar (despite low sequence identity) to bacterial mismatch-specific uracil-DNA glycosylases (MUGs) and to eukaryotic thymine-DNA glycosylates (TDGs), which use a base-flipping mechanism for the recognition of uracil and thymine^78,79^. We speculate that the MUG and TDG efficiencies will also be dictated in large part by substrate lesion DNA dynamics, though future studies are needed to test this hypothesis. Given the fundamental nature of genomic integrity, the implications of our studies are significant. That repair efficiencies, at least for UNG, are in large part dictated by DNA sequence deformability and flexibility could help explain the molecular mechanisms that underly fundamental observations in the fields of oncogenetics and cancer hotspots^80^, and evolutionary adaptation^81^. One particularly relevant context extends to the rapidly expanding field of base editing, where UNG and other glycosylases have been tethered to Cas9 nickases enabling precision DNA alterations with great potential for therapeutic intervention^82,83^. Ultimately, our data show a clear correlation between UNG activity and substrate flexibility that can be used to make predictions about the functional attributes of substrates, and may help rationalize sequence effects in base editing and other fields.

## Supplementary Information

Supplementary Information.

## Data Availability

The data that support the findings of this paper are available from the corresponding authors upon reasonable request. The NMR data used for fitting the *k*_*ex*_ are deposited under BMRB Entry ID 51612.
