# Supplementary material for: Uracil-DNA glycosylase efficiency is modulated by substrate rigidity
Source: Sci Rep. 2023 Mar 8;13:3915. doi: 10.1038/s41598-023-30620-0 (PMC9995336; doi:10.1038/s41598-023-30620-0)
Supplement: Supplementary file 1 — Supplementary Information. [file 41598_2023_30620_MOESM1_ESM.pdf]

Supporting Information: Uracil-DNA glycosylase efficiency is modulated by substrate rigidity

Paul B. Orndorff[1‡], Souvik Poddar[2,3‡], Aerial M. Owens[2,4‡], Nikita Kumari[2,3], Bryan T. Ugaz[2,3], Samrat Amin[5], Wade D. Van Horn[2,4,*], Arjan van der Vaart[1,*], Marcia Levitus[2,3,*]

[1] Department of Chemistry, University of South Florida, Tampa, Florida 33620, United States.
[2] School of Molecular Sciences, Arizona State University, 551 E. University Drive, Tempe, AZ, 85287, USA.
[3] The Biodesign Institute Center for Single Molecule Biophysics, Arizona State University, Tempe, AZ, 85287, USA.
[4] The Biodesign Institute Virginia G. Piper Center for Personalized Diagnostics, Arizona State University, Tempe, AZ, 85287, USA.
[5] Magnetic Resonance Research Center, Arizona State University, Tempe, AZ, 85287, USA.

* To whom correspondence should be addressed: **ML** Tel: +1-480-727-8586; Fax: +1-480-727-2378; Email: marcia.levitus@asu.edu, **AvdV** Tel: +1-813-974-8762; Fax: +1-813-974-3203; Email: avandervaart@usf.edu, **WVH**: +1-480-965-8322; Fax: +1-480-965-2747; Email: wade.van.horn@asu.edu

‡The first 3 authors should be regarded as joint First Authors.

Table of Contents

	Page
Structure of 2-Aminopurine incorporated into oligonucleotides.	S2
Sample kinetic run and a V_0 calculation and Fig. S1	S2
UNG assays with competing substrates	S3
Figure S2	S3
Figure S3	S4
Water inversion efficiency factor (E) and Fig. S4	S4-S5
Longitudinal relaxation of water (R_{1w}) and Fig. S5	S5
Imino proton longitudinal relaxation (R_{1n}) and exchange rates (k_{ex})	S6
Figure S6	S7
Figure S7	S8
Figure S8 (Step parameters for central $X_5U_6Y_7$ steps)	S9
Figure S9	S9
Figure S10 (Correlation of MD properties with k_{ex}.)	S10
Table S1(Fluorescence quantum yields)	S10
Table S2 (Fluorescence lifetimes)	S10
Table S3 (Fitting parameters for imino proton exchange rate measurement (k_{ex}))	S11
Table S4 (Michaelis-Menten parameters (K_m and V_{max}))	S12
Table S5 (α_0 values)	S12
Table S6 (MD properties)	S13

2AP substitution Structure of 2-Aminopurine (2AP) incorporated into oligonucleotides.

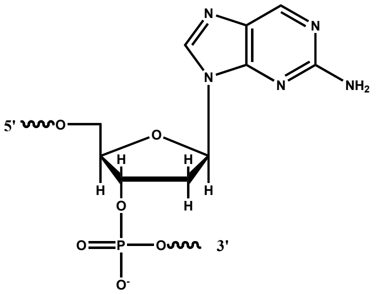

Sample kinetic run and a V_0 calculation

Figure S1. Fluorescence intensity (arbitrary units) vs time before and after the addition of UNG. The figure shows the results of a kinetic run using 1μM **1TA** DNA in 1x PBS buffer and 0.16 nM UNG. The measurement was interrupted briefly to add UNG and mix.

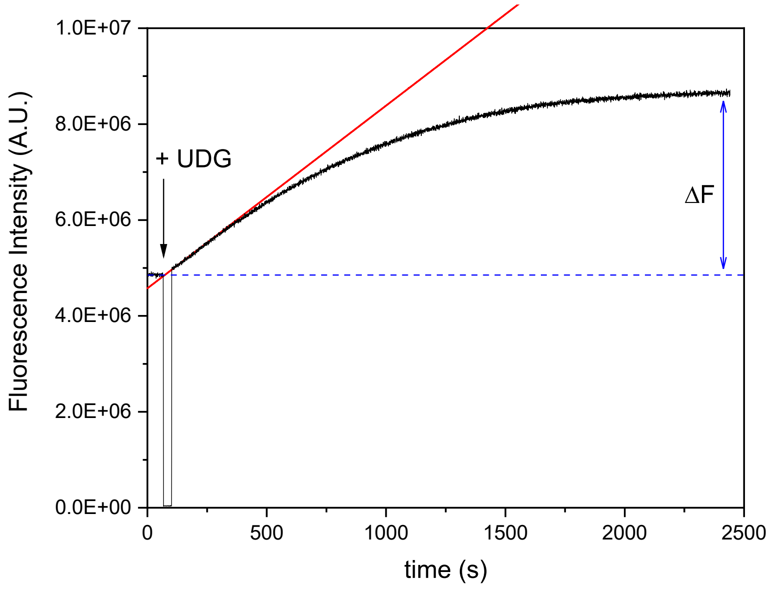

The initial velocity is calculated according to

$$V_0 = \frac{[S]_0}{\Delta F}\left(\frac{dF}{dt}\right)_0$$

Where $[S]_0 = 1.0 \times 10^{-6}$ M is the concentration of substrate, $\Delta F = 3.80 \times 10^6$ A.U is the difference between the final ($t \rightarrow \infty$) and initial Fluorescence intensity, and $\left(\frac{dF}{dt}\right)_0 = 3810\ A.U/s$ is the initial slope of the $F(t)$ vs t graph. A.U. denotes arbitrary units. A value of $V_0 = 1.0 \times 10^{-9}\mathrm{M}.s^{-1}$ was calculated using data measured in this experiment.

UNG assays with competing substrates

Kinetic assays were carried out with two competing substrates in solution to obtain relative catalytic efficiencies. Two experiments were performed for each pair of substrates. In the first, substrate *x* contained 2AP opposite to the uracil, while substrate *y* contained a canonical adenine. The velocity measured in this experiment is V_x, the rate of uracil removal from substrate *x* when the enzyme competes for substrates *x* and *y*. The second experiment was carried out with 2AP-labeled substrate *y* and unlabeled *x*, from which V_y was calculated. The ratio V_x/V_y equals the ratio of the catalytic efficiencies, $\frac{(k_{cat}^x/K_M^x)}{(k_{cat}^y/K_M^y)}$, when the concentrations of substrates *x* and *y* are equal and kept constant in both experiments.[1,2] Competition experiments were carried out for three pairs of substrates (1TA/1AT, 2TA/2AT, and 1TA/2TA, see table 1) with solutions containing 0.3 μM of each substrate and 0.16 nM UNG.

Values of $\frac{(k_{cat}^x/K_M^x)}{(k_{cat}^y/K_M^y)}$ obtained in this way are $V_{1TA}/V_{1AT} = 1.65$, $V_{2TA}/V_{2AT} = 1.59$, and $V_{1TA}/V_{2TA} = 1.44$. These values agree with the $\frac{(k_{cat}^x/K_M^x)}{(k_{cat}^y/K_M^y)}$ ratios calculated from the k_{cat} and K_m values obtained from the Michaelis-Menten fits within experimental error. The first two pairs (1TA/1AT and 2TA/2AT) confirm that UNG's specificity is greater for uracil in a TUA context compared to AUT. The third pair (1TA/2TA) indicates that substrate specificity is not solely determined by the flanking bases (see also Fig.1, inset).

Figure S2 Two-dimensional ^{1}H, ^{1}H - Nuclear Overhauser Effect Spectroscopy (NOESY) experiments were measured at 20 °C. Resulting 2D spectra were used to assign imino protons using traditional "backbone-walking" methods.[3,4]

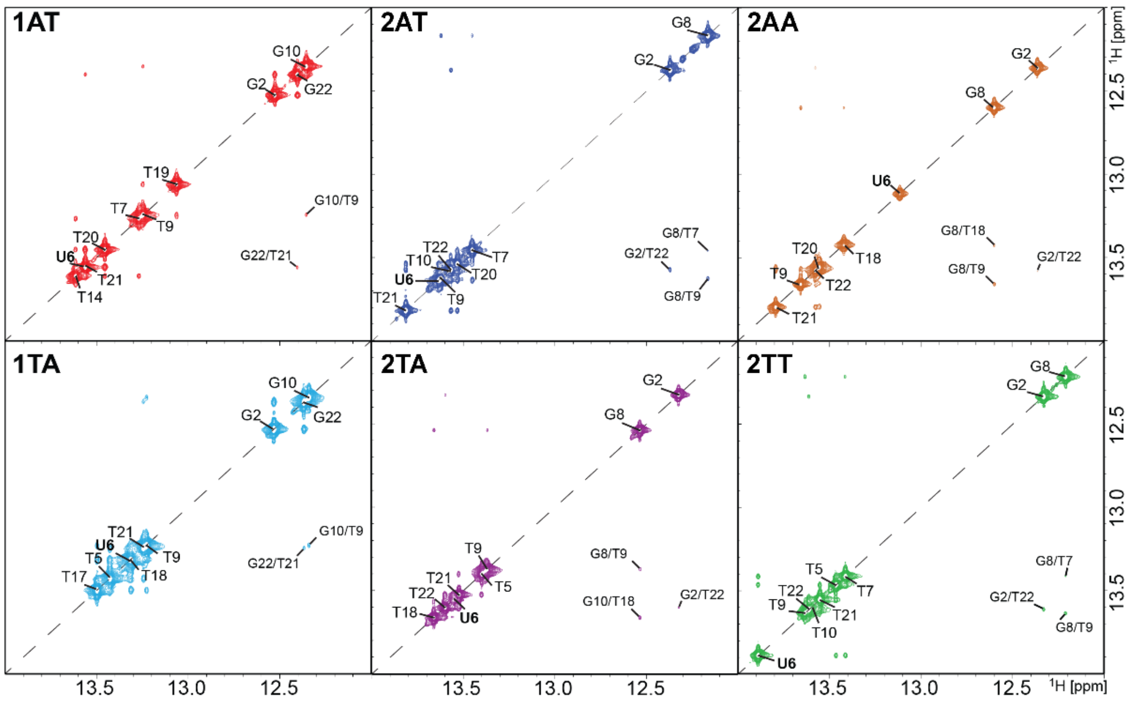

Figure S3 A ^{1}H one-dimensional representation of the imino assignments determined from NOESY spectra in Fig. S2. Accompanying sequences include assigned imino bases in **bold**. All ^{1}H-1D experiments were measured at 20°C.

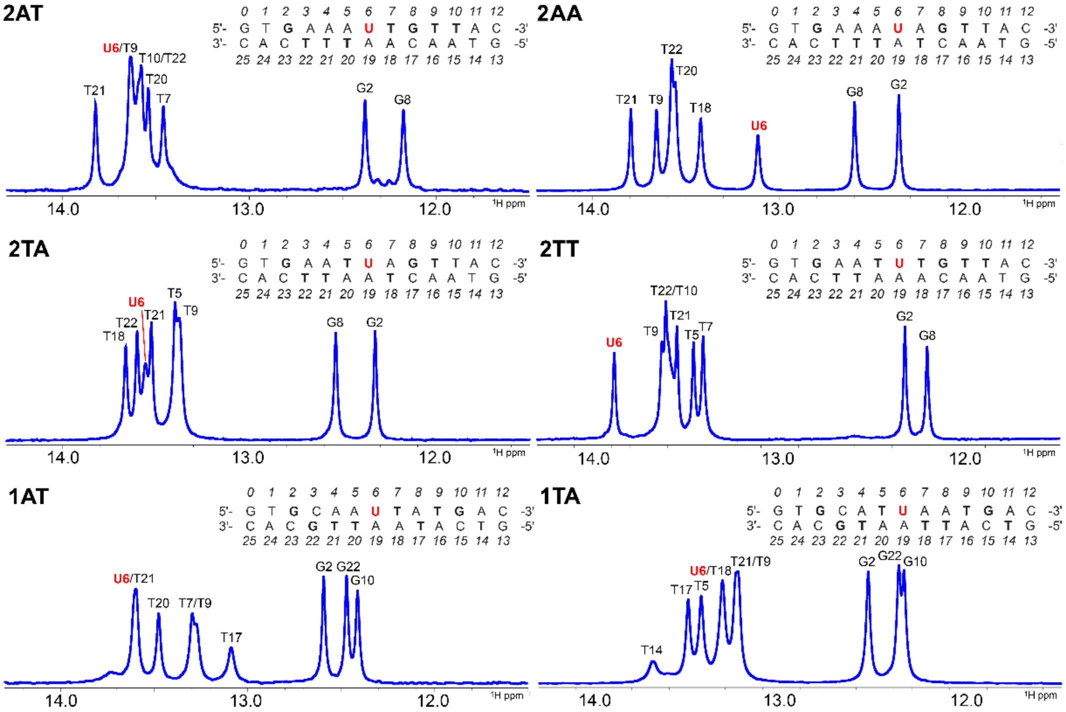

Water inversion efficiency factor (E)

The water inversion efficiency factor *(E)* was determined in a 3 mm NMR tube on both the 600 MHz (Fig. S4A) and 850 MHz (Fig. S42B) instruments. The peak areas for each water signal were determined by manually phasing the spectra in Topspin 4.1 and MestReNova 14.2, respectively, and measuring the peak integrals in an overlayed spectrum. The measured peak areas were applied to the following equation

$$E = 1 - \frac{W_{inv}}{W_{eq}} \qquad \text{Eq. S1}$$

where W_{inv} is the peak area of the signal after inversion and W_{eq} is the peak area of the signal without inversion. The resulting *E* factor represents the efficiency of the selective pulse inversion, where *E* = 2 indicates total inversion efficiency. Previously reported *E* factors range from 1.90-2.00.[5] However, since the efficiency is mostly dependent on instrument hardware, these reference values could be inconstant. The measured *E* factor for the 600 MHz and 850 MHz were 1.905 and 1.943, respectively (Fig. S4C-D).

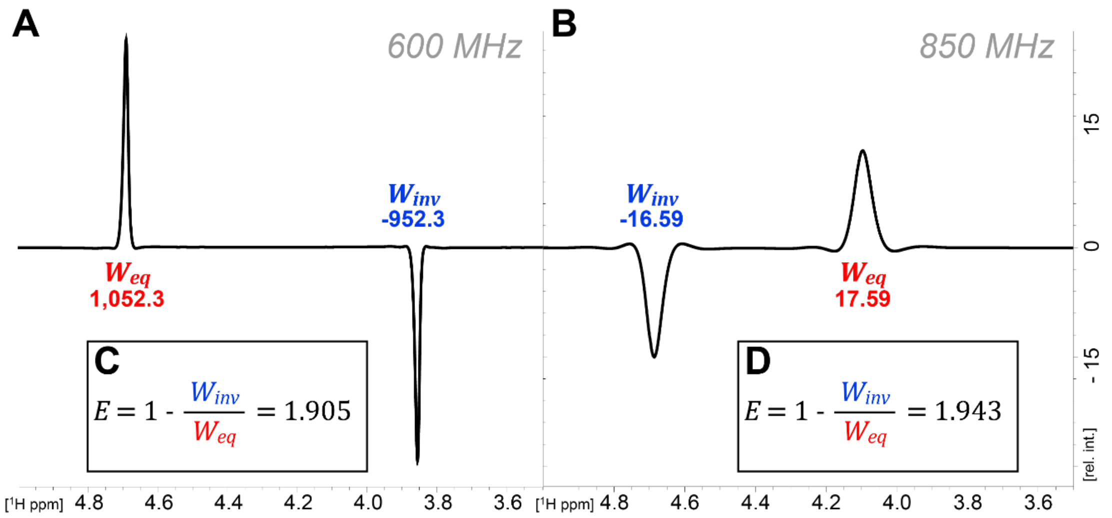

Fig. S4. Determination of the water inversion efficiency factor (E). A) The water signal peak area with (W_{inv}, blue) and without (W_{eq}, red) a DANTE inversion pulse[3,4] measured on the 600 MHz and B) the 850 MHz instruments. Spectra are overlayed and referencing is shifted 500 Hz for each. C) Applying Equation S1, the measured E factor for the 600 MHz instrument is 1.905 and for D) the 850 MHz instrument it is 1.943.

Longitudinal relaxation of water (R_{1w})

The longitudinal relaxation of water (R_{1w}) was measured on the 600 MHz instrument in a 3 mm NMR tube using a list of 24 variable time delays ranging from 1 ms to 18 s and a relaxation delay of 30 s. Determination of R_{1w} was initially completed using the TopSpin T1/T2 Module and fitting to the equation

$$A = \alpha + \beta e^{-tR_{1w}} \quad \text{Eq. S2}$$

where A is the area of the water signal peak, t is the relaxation delay time, and α and β are constants. The TopSpin module resulted in a R_{1w} of 0.5051. This value was verified in MATLAB R2019b (Mathworks) Curve Fitting Tool to be 0.504 ± 0.012 (Fig. S5). Multiple trials were performed on the 600 MHz and 850 MHz magnets, which resulted in a working range of 0.4985 to 0.5102 for the R_{1w} parameter in k_{ex} fitting.

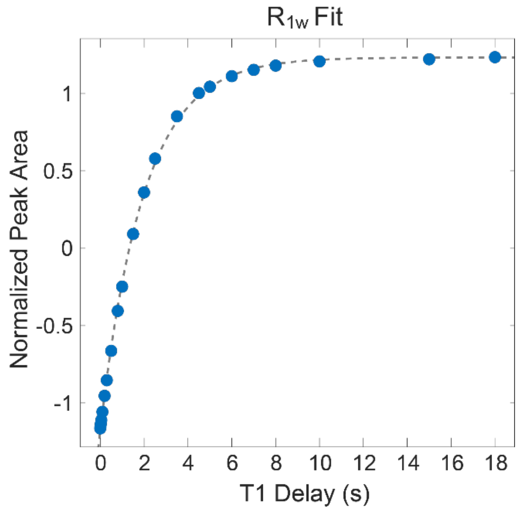

Fig. S5. Determination of the water longitudinal relaxation rate (R_{1w}). The water signal peak areas (blue) as a function of T1 delay was fit to Equation S2 (gray, dashed line) and rendered a R_{1w} value of 0.5051.

Imino proton longitudinal relaxation (R_{1n}) and exchange rates (k_{ex})

The longitudinal relaxation of water (R_{1n}) was initially measured on the 850 MHz instrument in a 3 mm NMR tube using a list of 24 variable time delays ranging from 1 ms to 15 s. However, imino proton peaks in some sequences exhibited low resolution and S/N despite the high-field instrument. For these sequences, data was collected on the 600 MHz instrument where resolution and S/N significantly improved, presumably because of more favorable relaxation properties. Previously described pseudo two-dimensional experiments[6] for the imino proton R_{1n} and imino proton exchange rate (k_{ex}) were measured on the same instrument for each sequence.

The respective spectra were processed and phased in TopSpin 4.1. The data were baseline corrected and fit with MATLAB R2019b (MathWorks) using nonlinear least squares fit (Fig. S6A-D). The R_{1n} was determined by fitting the individual imino proton peaks areas to the equation:

$$A = \alpha + \beta e^{-tR_{1n}} \qquad \text{Eq. S3}$$

where A is the area under the peak, t is the relaxation delay time, and α and β are constants (Fig. S6E). The error of the fit was used as the variable bounds for the R_{1n} parameter in fitting for k_{ex}. The (k_{ex}) was determined by fitting the individual imino proton peak areas to Equation 1 (main manuscript) (Fig. S6F). The R_{1w} and R_{1n} parameters for fitting k_{ex} were permitted to float within the error bounds previously determined in the respective fits, whereas the E parameter was fixed for data collected on the 600 MHz and 850 MHz, respectively (Table S1). The final k_{ex} for each imino proton was reported with the error of the fit. Associated NMR data are deposited under BMRB Entry ID 51612.

Figure S6 Example of determining the imino proton longitudinal relaxation (R_{1n}) and exchange (k_{ex}) rates from 1AT. A) Spectra processed and phased in TopSpin 4.1 were baseline corrected in MATLAB R2019b (MathWorks). B) Peaks were fit to the new baseline with a Lorentzian function and C) individual peaks were fit based on predetermined peak positions. D) Resulting residuals of individual peak fitting. E) R_{1n} fitting of the 1AT G2 imino proton peak areas to Equation S3. F) k_{ex} fitting of the 1AT G2 imino proton peak areas to Equation 1 (main manuscript).

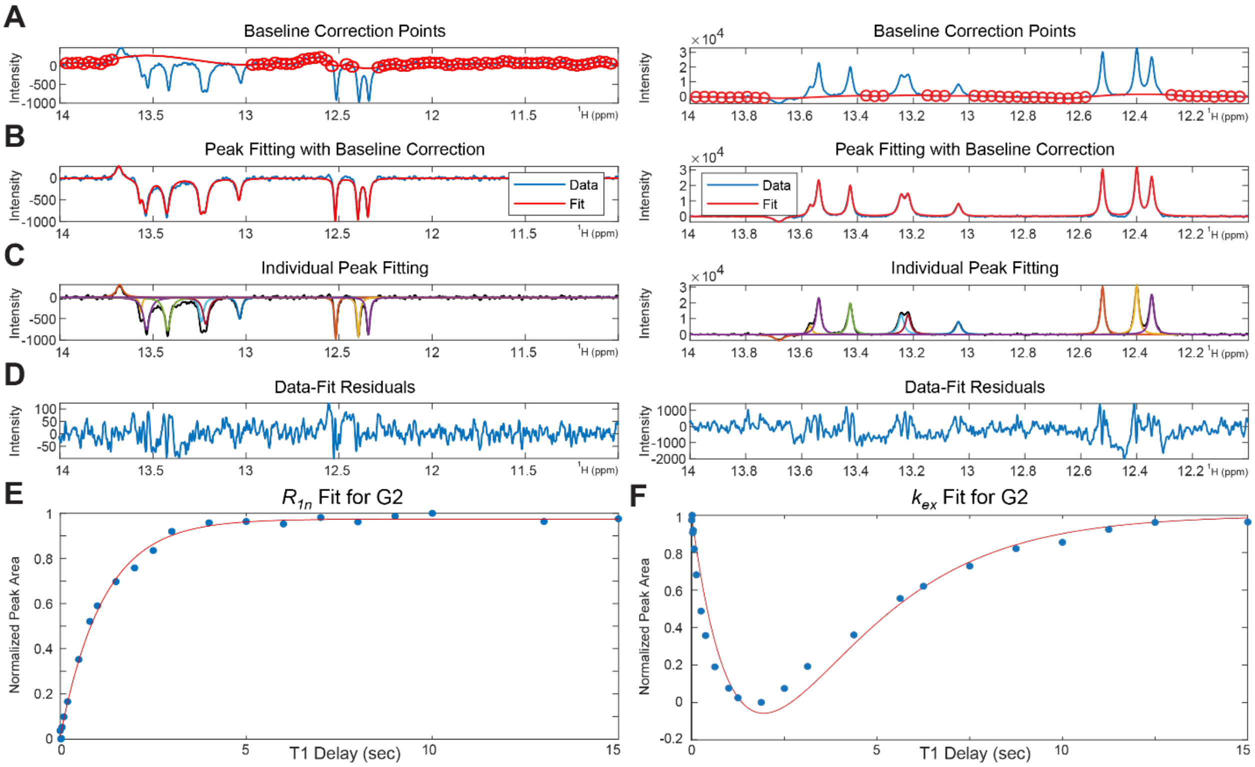

Figure S7. Experimental initial rates and Michaelis-Menten plots for samples 4TA, 2TA, 1AT, 3AT, 1AA, 2AA, 1TT and 2TT. The corresponding graphs for samples 1TA and 2AT are shown in Fig. 1. Michalis-Menten parameters are shown in Table S2. UNG concentration was 0.16 nM in all cases.

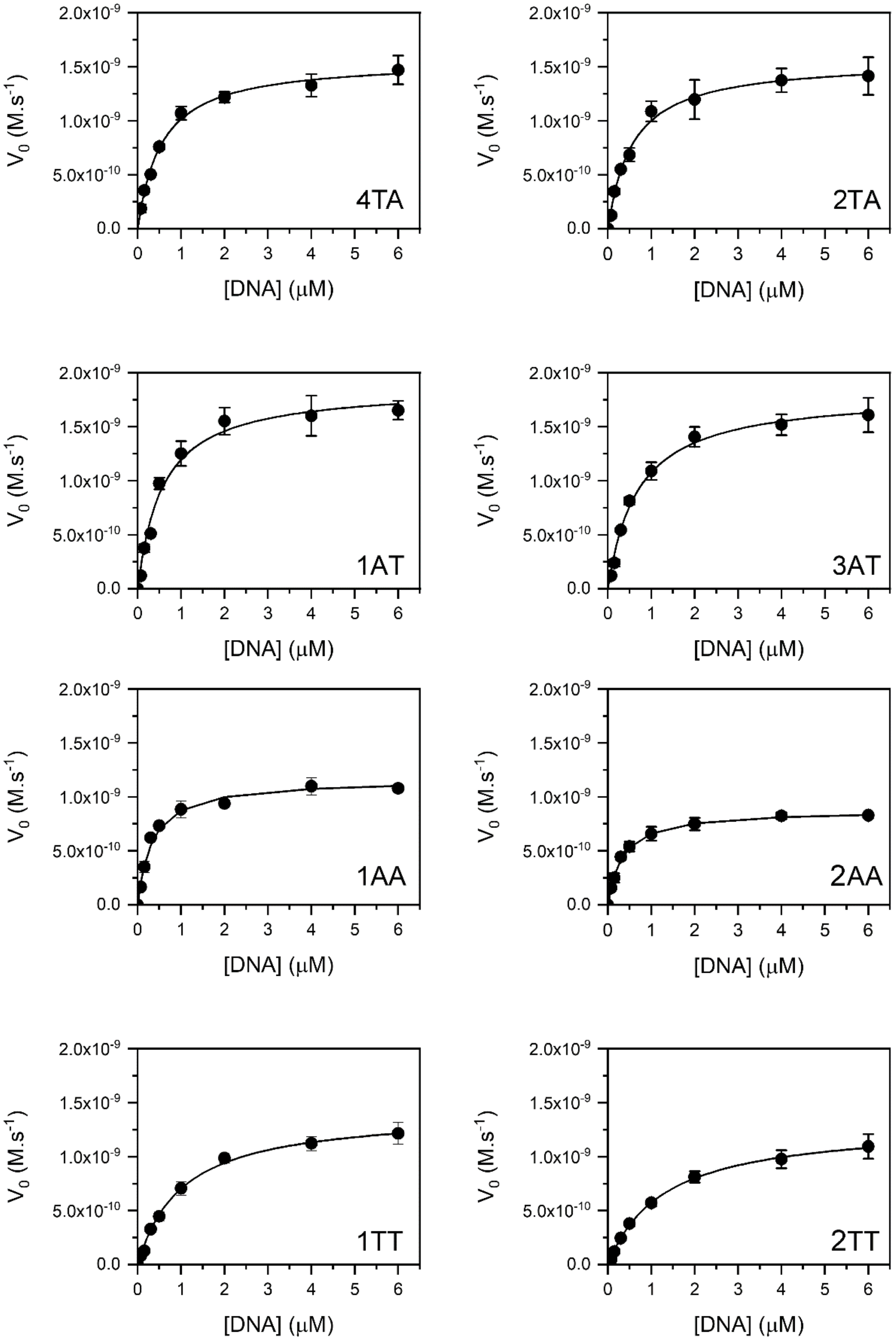

Figure S8 Step parameters for central $X_5U_6Y_7$ steps. Averages and standard deviations over all sequences and all simulations.

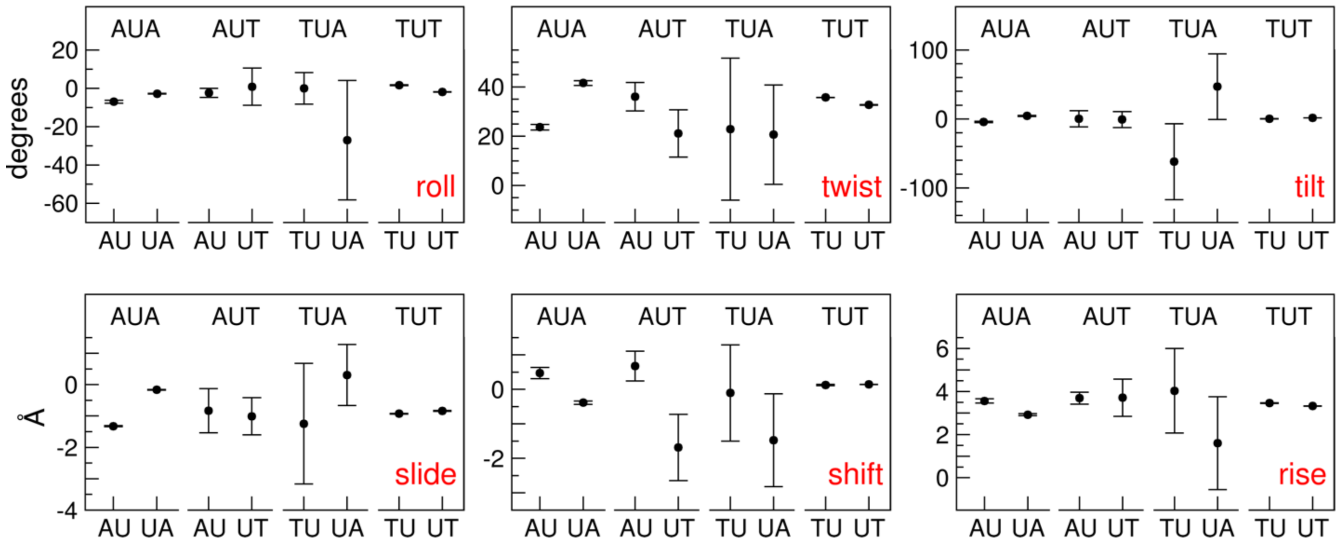

Figure S9 Michaelis-Menten parameters (K_m and V_{max}) from Table S2 plotted against α_0 values from table S5. Results indicate that the values of Km, but not k_{cat}, are dictated by the mechanical properties of the substrate.

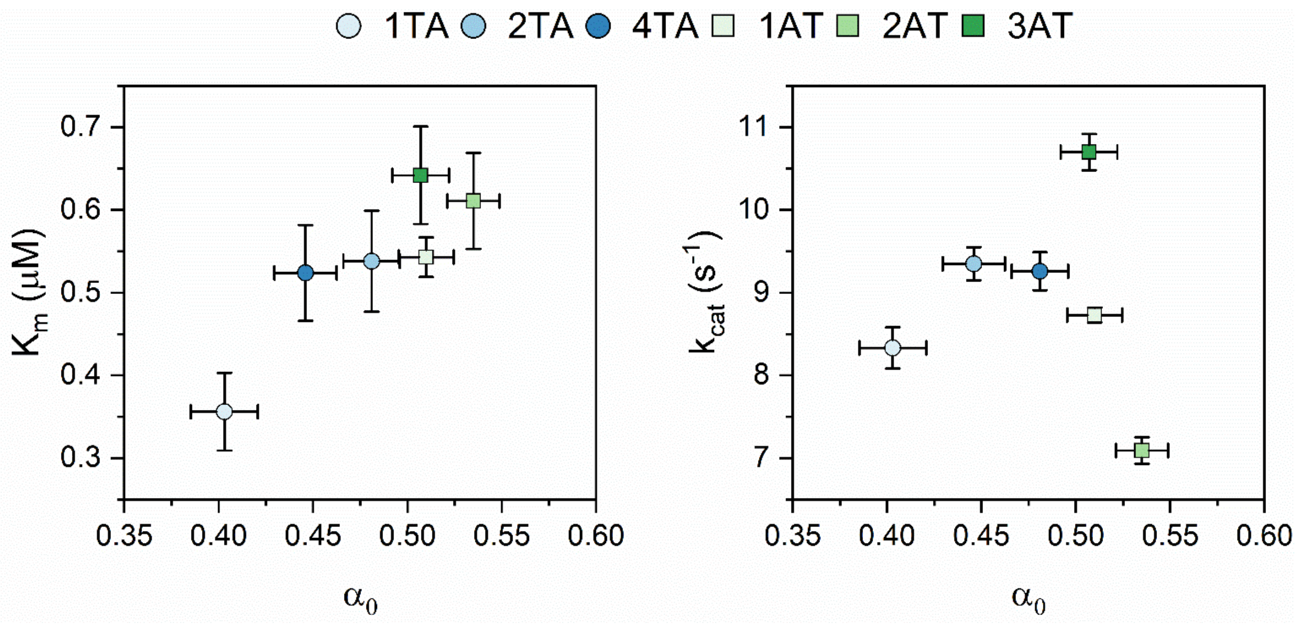

Figure S10 Correlation of MD properties with k_{ex}. A) Bending persistence length (in Å), B) torsional persistence length (in Å), C) standard deviation of the bending angle (in degrees). Averages and standard deviations over 3 MD replicas; k_{ex} in s^{-1}. Red lines show linear regression of all data with correlation coefficients of -0.889 (A), -0.739 (B), and 0.788 (C); blue lines linear regressions excluding the 4TA sequence with correlation coefficients of -0.504 (A), 0.054 (B), and 0.239 (C).

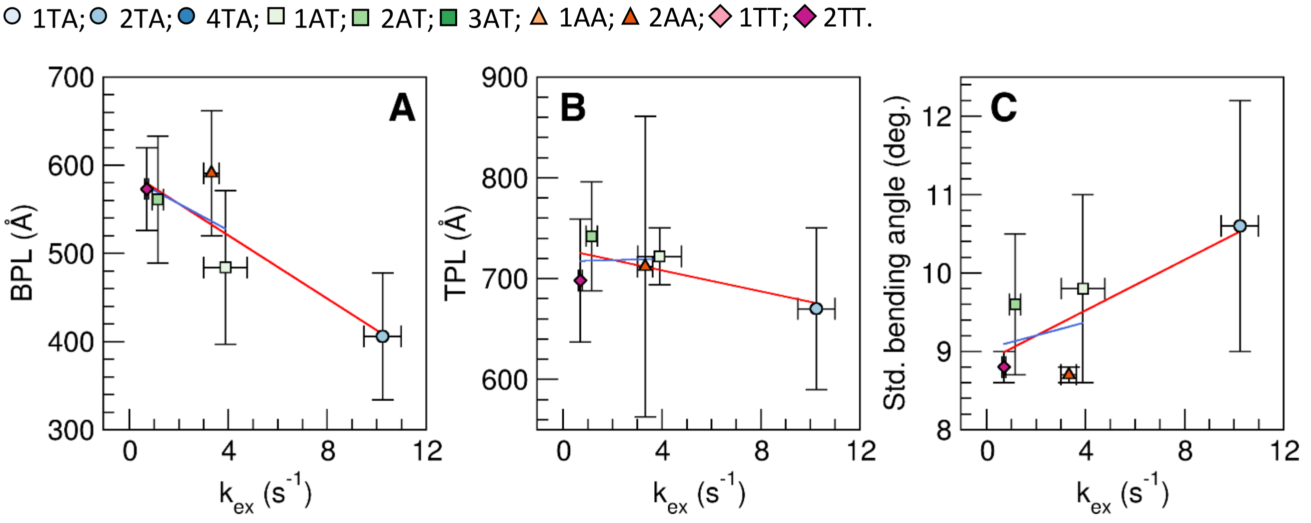

Table S1 Fluorescence quantum yields (ϕ) were determined as described in Materials and Methods. Values are the average of at least 4 determinations. All standard deviations are 3% or less.

Sample	1TA	1AT	2TA	2AT	3AT	4TA
ϕ	0.00383	0.0103	0.00356	0.00606	0.00981	0.00308

Table S2 Results of two independent TCSPC experiments. Time-resolved fluorescence intensity decays were acquired and fitted as discussed in Materials and Methods. $F(t) = \sum_{i=1}^{4} \alpha_i e^{-t/\tau_i}$, $\sum_{i=1}^{4} \alpha_i = 1$, $\langle\tau\rangle = \sum_{n=1}^{4} \alpha_i \tau_i$

Experiment 1

Sample	α_1	τ_1 (ns)	α_2	τ_2 (ns)	α_3	τ_3 (ns)	α_4	τ_4 (ns)	$\langle\tau\rangle$ (ns)
1TA	0.709	0.0339	0.278	0.117	0.00937	1.46	0.00394	5.83	0.0932
1AT	0.402	0.0662	0.495	0.312	0.0969	0.999	0.00624	6.09	0.316
2TA	0.676	0.0387	0.310	0.128	0.00970	1.66	0.00377	4.93	0.100
2AT	0.593	0.0644	0.375	0.244	0.0253	1.082	0.00607	5.95	0.193
3AT	0.381	0.0780	0.557	0.307	0.0556	1.05	0.00629	5.55	0.294
4TA	0.873	0.0326	0.116	0.144	0.00686	1.87	0.00408	5.92	0.0821

Experiment 2

Sample	α_1	τ_1 (ns)	α_2	τ_2 (ns)	α_3	τ_3 (ns)	α_4	τ_4 (ns)	$\langle\tau\rangle$ (ns)
1TA	0.725	0.0366	0.263	0.123	0.00905	1.68	0.00365	6.98	0.0995
1AT	0.420	0.0693	0.467	0.308	0.107	0.919	0.00642	6.68	0.315
2TA	0.675	0.0386	0.311	0.125	0.0106	1.69	0.00391	5.85	0.106
2AT	0.574	0.0653	0.397	0.245	0.0230	1.20	0.00529	6.60	0.197
3AT	0.384	0.0854	0.549	0.310	0.0603	0.982	0.00655	6.13	0.302
4TA	0.849	0.0328	0.140	0.122	0.00769	1.53	0.00405	6.59	0.0833

Table S3. Fitting parameters for imino proton exchange rate measurement (k_{ex})

2AT						
Imino	k_{ex} (s^{-1})	Error	R_{1n} (s^{-1})	Error	^{1}H ppm	σ
G2	1.03	0.15	0.819	0.102	12.3566	0.0001
T22	0.50	0.16	0.294	0.281	13.5537	0.0045
T21	0.81	0.06	0.626	0.077	13.7983	0.0001
T20	0.52	0.12	0.252	0.122	13.5161	0.0001
**U6**	1.15	0.22	1.197	0.449	13.6164	0.0011
T7	0.57	0.09	0.352	0.161	13.4360	0.0003
G8	1.02	0.12	0.892	0.101	12.1508	0.0001
T9	7.02	2.25	9.593	3.922	13.6046	0.0044
2TA						
Imino	k_{ex} (s^{-1})	Error	R_{1n} (s^{-1})	Error	^{1}H ppm	σ
G2	1.16	0.11	1.040	0.097	12.3079	0.0001
T22	1.19	0.20	1.065	0.321	13.5813	0.0003
T21	0.99	0.15	0.844	0.275	13.5051	0.0002
T5	1.34	0.30	1.238	0.215	13.3776	0.0004
**U6**	10.24	0.75	14.923	1.838	13.5373	0.0067
T18	1.68	0.15	1.827	0.298	13.6429	0.0003
G8	0.94	0.10	0.774	0.050	12.5195	0.0002
T9	1.60	0.19	1.646	0.262	13.3521	0.0013
2AA						
Imino	k_{ex} (s^{-1})	Error	R_{1n} (s^{-1})	Error	^{1}H ppm	σ
G2	1.06	0.10	0.884	0.110	12.4062	0.0001
T22	2.93	1.11	3.569	2.458	13.6130	0.0061
T21	0.82	0.06	0.603	0.057	13.8232	0.0001
T20	1.17	0.22	1.096	0.413	13.5661	0.0007
**U6**	3.32	0.31	4.285	0.991	13.1387	0.0002
T18	1.91	0.18	2.109	0.755	13.4357	0.0022
G8	0.79	0.06	0.580	0.020	12.6260	0.0001
T9	1.09	0.17	1.009	0.177	13.6849	0.0003
2TT						
Imino	k_{ex} (s^{-1})	Error	R_{1n} (s^{-1})	Error	^{1}H ppm	σ
G2	0.85	0.09	0.618	0.024	12.3291	0.0001
T22	0.89	0.17	0.718	0.123	13.6090	0.0017
T21	0.67	0.09	0.438	0.069	13.5494	0.0002
T5	0.67	0.04	0.460	0.083	13.4608	0.0000
**U6**	0.70	0.08	0.475	0.133	13.8850	0.0001
T7	0.78	0.27	0.569	0.044	13.4095	0.0005
G8	0.80	0.09	0.564	0.026	12.2099	0.0001
T9	1.49	0.17	1.593	0.363	13.6282	0.0058
1AT						
Imino	k_{ex} (s^{-1})	Error	R_{1n} (s^{-1})	Error	^{1}H ppm	σ

G2	1.02	0.11	0.885	0.089	12.5217	0.0002
G22	0.89	0.11	0.765	0.060	12.3989	0.0003
T21	0.63	0.14	0.342	0.081	13.5386	0.0006
T20	1.12	0.15	1.031	0.105	13.4259	0.0002
**U6**	3.90	0.88	5.071	0.218	13.5732	0.0070
T7	1.32	0.12	1.246	0.247	13.2430	0.0003
T17	1.56	0.15	1.655	0.326	13.0378	0.0003
T9	1.08	0.07	1.017	0.102	13.2189	0.0003
G10	1.29	0.13	1.235	0.124	12.3455	0.0002

Table S4 Michaelis-Menten parameters (Km and Vmax) where obtained by fitting the data shown in Figs. 1 and S7 to the following equation: $V_0 = \frac{V_{max}[S]}{K_m+[S]}$. The catalytic constant, k_{cat}, was calculated as $V_{max}/[E]$, where [E], the enzyme concentration, was calculated using the specific activity provided by the manufacturer (100 U/μg). Errors in Table S2 are calculated from the standard errors of the fitting parameters. Fitting was done using the Lavenberg Marquardt iteration algorithm using the reciprocal of the variances of each point as weights.

Sample	K_M (μM)	k_{cat} (s^{-1})	$\frac{k_{cat}}{K_M}$ (M^{-1} s^{-1})
1TA	0.356 ± 0.047	8.33 ± 0.25	(2.34 ± 0.32) × 10^7
1AT	0.543 ± 0.024	8.731 ± 0.090	(1.608 ± 0.073) × 10^7
1AA	0.321 ± 0.029	6.99 ± 0.15	(2.18 ± 0.20) × 10^7
1TT	1.016 ± 0.057	8.53 ± 0.13	(0.840 ± 0.049) × 10^7
2TA	0.538 ± 0.061	9.26 ± 0.23	(1.72 ± 0.20) × 10^7
2AT	0.611 ± 0.058	7.09 ± 0.16	(1.16 ± 0.11) × 10^7
2AA	0.332 ± 0.018	5.282 ± 0.072	(1.589 ± 0.088) × 10^7
2TT	1.286 ± 0.061	7.89 ± 0.10	(0.614 ± 0.030) × 10^7
3AT	0.642 ± 0.059	10.7 ± 0.22	(1.67 ± 0.15) × 10^7
4TA	0.524 ± 0.058	9.35 ± 0.20	(1.78 ± 0.22) × 10^7

Table S5 Fractional concentration of highly stacked 2AP (α_0). Values of $\alpha_0$were calculated as $\alpha_0 = 1 - \frac{\tau_{2AP}}{\langle\tau\rangle}\frac{\phi}{\phi_{2AP}}$, using the values of Tables S3 and S4 and $\phi_{2AP} = 0.68$ and $\tau_{2AP} = 10.2$ ns (see Materials and Methods)

Sample	1TA	1AT	2TA	2AT	3AT	4TA
α_0	0.403 ± 0.018	0.510 ± 0.015	0.481 ± 0.015	0.535 ± 0.014	0.507 ± 0.015	0.446 ± 0.017

Table S6 MD properties; averages and standard deviations over 3 MD replicas.

Sequence	Bending persistence length (Å)	Torsional persistence length (Å)	Standard deviation of bending angle (degrees)
1TA	316 ± 54	617 ± 31	12.2 ± 1.2
2TA	406 ± 72	670 ± 80	10.6 ± 1.6
4TA	424 ± 55	633 ± 145	10.5 ± 1.2
1AT	484 ± 87	722 ± 28	9.8 ± 1.2
2AT	561 ± 72	742 ± 54	9.6 ± 0.9
3AT	444 ± 18	654 ± 40	10.1 ± 0.7
1AA	560 ± 21	719 ± 121	8.9 ± 0.4
2AA	591 ± 71	712 ± 149	8.7 ± 0.1
1TT	564 ± 57	734 ± 9	8.8 ± 0.2
2TT	573 ± 47	698 ± 61	8.8 ± 0.2

References

(1) Eisenthal, R., Danson, M. J. & Hough, D. W. Catalytic efficiency and kcat/KM: a useful comparator? Trends Biotechnol **2007** 25, 247-249

(2) Lorsch, J. R. in Methods in Enzymology Vol. 536 (ed Jon Lorsch) 3-15 (Academic Press, **2014**).

(3) Fürtig, B.; Richter, C.; Wöhnert, J.; Schwalbe, H. NMR Spectroscopy of RNA. *ChemBioChem* **2003**, *4*, 936-962.

(4) Weiss, M. A.; Patel, D. J.; Sauer, R. T.; Karplus, M. Two-dimensional 1H NMR study of the lambda operator site OL1: a sequential assignment strategy and its application. *Proc. Natl. Acad. Sci. U.S.A.* **1984**, *81*, 130-134.

(5) Crenshaw, C. M.; Wade, J. E.; Arthanari, H.; Frueh, D.; Lane, B. F.; Núñez, M. E. Hidden in plain sight: subtle effects of the 8-oxoguanine lesion on the structure, dynamics, and thermodynamics of a 15-base pair oligodeoxynucleotide duplex. *Biochemistry* **2011**, *50*, 8463-8477.

(6) Szulik, M. W.; Voehler, M.; Stone, M. P. NMR analysis of base-pair opening kinetics in DNA. *Curr Protoc Nucleic Acid Chem* **2014**, *59*, 7.20.21-27.20.18.
